# Comparison of autism domains across thirty rare variant genotypes

**DOI:** 10.1016/j.ebiom.2024.105521

**Published:** 2025-01-31

**Authors:** Nabila M.H. Ali, Samuel J.R.A. Chawner, Leila Kushan-Wells, Carrie E. Bearden, Jennifer Gladys Mulle, Rebecca M. Pollak, Raquel E. Gur, Wendy K. Chung, IMAGINE ID Consortium, Harriet Housby, Irene Lee, David Skuse, Jeanne Wolstencroft, William Mandy, Spiros Denaxas, Kate Baker, Lucy Raymond, Marianne van den Bree, Samuel Chawner, Jeremy Hall, Peter Holmans, Josh Hope-Bell, Danielle Le Roux, Sally Morrin, Michael Owen, Shreeya Sivakumar, Michael J. Owen, Marianne B.M. van den Bree

**Affiliations:** a Division of Psychological Medicine and Clinical Neurosciences, Centre for Neuropsychiatric Genetics and Genomics, Cardiff University, UK; b Departments of Psychiatry and Behavioral Sciences and Psychology, Semel Institute for Neuroscience and Human Behavior, University of California Los Angeles, USA; c Department of Psychiatry, Robert Wood Johnson School of Medicine, Rutgers University, USA; d Center for Advanced Biotechnology and Medicine, Rutgers University, USA; e Department of Psychiatry, Neurodevelopment & Psychosis Section, Perelman School of Medicine, University of Pennsylvania, Philadelphia, PA, USA; f Department of Pediatrics, Boston Children's Hospital and Harvard Medical School, Boston, MA, USA

**Keywords:** Autism, Rare genetic variants, Copy number variants (CNVs), Single gene variants (SGVs)

## Abstract

**Background:**

A number of Neurodevelopmental risk Copy Number Variants (ND-CNVs) and Single Gene Variants (SGVs) are strongly linked to elevated likelihood of autism. However, few studies have examined the impact on autism phenotypes across a wide range of rare variant genotypes.

**Methods:**

This study compared Social Communication Questionnaire (SCQ) scores (total and subdomains: social, communication, repetitive behaviour) in 1314 young people with one of thirty rare variant genotypes (15 ND-CNVs; n = 1005, 9.2 ± 3.5 years and 15 SGVs; n = 309, 8.3 ± 4.0 years). Comparisons were also conducted with young people without known genetic conditions (controls; n = 460, 10.6 ± 3.4 years) and with idiopathic autism (n = 480, 8.6 ± 3.2 years).

**Findings:**

The prevalence of indicative autism (SCQ ≥ 22) was higher in those with a rare variant genotype compared to controls (32% vs 2%; OR = 43.1, CI = 6.6–282.2, p < 0.001) and in those with SGVs compared to ND-CNVs (53% vs 25%; OR = 4.00, CI = 2.2–7.3, p = 0.002). The prevalence of indicative autism varied considerably across the 30 rare variant genotypes (range 10–85%). SGVs were associated with greater impairment in total, social, communication and repetitive behaviour subdomains than ND-CNVs. However, genotype explained limited variation in these scores (*η*^2^ between 11.8 and 21.4%), indicating more convergence than divergence in autism phenotype across rare variant genotypes. Comparisons with young people with idiopathic autism indicated no differences compared to those with ND-CNVs, whereas those with SGVs showed greater communication and less repetitive behaviour.

**Interpretation:**

The likelihood of autism was higher across all rare variant genotypes, with individuals with SGVs showing higher prevalence and greater impairment compared to those with ND-CNVs. Despite subdomain-specific patterns, there was no strong evidence for specific genotype-phenotype associations. This suggests that rare variant genotypes alone may have limited predictive value for autism phenotypes and that other factors like polygenic risk and the environment are likely to play a role. Further research is needed in order to understand these influences, improve risk prediction and inform genetic counselling and interventions.

**Funding:**

This work was funded by the Tackling Multimorbidity at Scale Strategic Priorities Fund programme (MR/W014416/1) (van den Bree) delivered by the Medical Research Council and the National Institute for Health Research in partnership with the Economic and Social Research Council and in collaboration with the Engineering and Physical Sciences Research Council. NIMH U01 MH119738-01 (van den Bree), IMAGINE study (Medical Research Council UK: MR/T033045/1; MR/N022572/1; and MR/L011166/1) (van den Bree) and Medical Research Council UK Centre Grant (MR/L010305/1) (Owen). SJRAC is funded by a Medical Research Foundation Fellowship (MRF-058-0015-F-CHAW). We would also like to acknowledge NIH 1R01MH110701-01A1 (PI Mulle), U01MH119736 (CEB), *R21MH116473 (CEB),* and R01MH085953 (CEB), and the Simons Foundation (SFARI Explorer Award to CEB).

**Research in context**

**Evidence before this study**

Autism is characterised by impairments in social communication, restricted interests, and repetitive behaviour. It is commonly associated with other neurodevelopmental disorders, such as attention deficit hyperactivity disorder (ADHD) and learning disabilities. Genetic factors play a considerable role in autism, with rare Neurodevelopmental risk Copy Number Variants (ND-CNVs) and Single Gene Variants (SGVs) exerting larger effects than common genetic variants. We searched titles and abstracts in PubMed published until September 2024 for studies in English that included the terms “autism”, “copy number variants”, and “single gene variants”, as well as each of the 30 rare variant genotypes that are part of our study. We found no clear evidence addressing the question of whether specific rare genetic variants were associated with specific autism subdomains. It is also not clear from the current literature whether autism in individuals with rare variant genotypes differs from idiopathic autism (i.e., with no known genetic origin).

**Added value of this study**

The majority of studies examining genotype-phenotype relationships within autism to date have included small samples and limited numbers of rare variant genotypes. Our international collaboration brought together a relatively large sample of young people (n = 2243) comprising 1314 with one of 30 rare variant genotypes, 480 with idiopathic autism, and 460 neurotypical individuals without known genetic conditions (controls). Indicative autism prevalence and subdomain scores were compared across rare variant genotypes and differences with individuals with idiopathic autism were investigated using the social communication questionnaire (SCQ).

Having a rare variant genotype was associated with a 43-fold increased likelihood of indicative autism. Individuals with SGVs had a higher prevalence of indicative autism than those with ND-CNVs. The prevalence of indicative autism varied considerably between rare variant genotypes (ranging from 10% to 85%). Similarly, considerable differences were found in autism total and subdomain scores across rare variant genotypes. Comparison of autism subdomains between those with rare variant genotypes and those with idiopathic autism indicated comparable profiles for those with ND-CNVs, whereas those with SGVs showed greater impairment in the communication subdomain and less impairment in the repetitive behaviour subdomain. An individual's specific variant genotype explained between 11.8 and 21.4% of the variation in indicative autism total and subdomain scores.

**Implications of all the available evidence**

Rare variant genotypes are strongly associated with autism, with large variations in indicative autism prevalence and subdomain scores. Although rare variant genotypes showed subdomain-specific patterns, we did not find strong evidence of specific genotype-phenotype associations. Rather, our findings indicate a greater degree of convergence than divergence in autism prevalence and subdomain scores across rare variant genotypes. This suggests that rare variant genotypes alone may have limited predictive value for autism phenotypes and that other factors like polygenic risk and aspects of an individual's environment will also need to be taken into account to better understand the development of autism. Further study of the role of these factors in the development of autism in young people with rare variants will be important for the delivery of improved counselling and intervention services.

## Introduction

Individuals with autism experience persistent impairment in social interaction and social communication, as well as an increase in restricted interests and repetitive behaviour.^1^ The genetic architecture underlying autism is complex, involving hundreds of common variants of small effect size^2^^,^^3^ and a range of rare Copy Number Variants (CNVs) and Single Gene Variants (SGVs) of relatively large effect size.^4^, ^5^, ^6^, ^7^ CNVs involve deletions or duplications of chromosomal segments larger than 1000 base pairs and are present in ∼5–10% of individuals with autism.^8^, ^9^, ^10^ Recently, the term Neurodevelopmental risk CNVs (ND-CNVs) has been used to refer to recurrent reciprocal CNVs associated with a high risk of neurodevelopmental disorder.^11^, ^12^, ^13^ Rare SGVs, disrupting specific genes, also contribute to autism aetiology and are estimated to be present in around ∼10–30% of individuals with autism.^10^^,^^14^^,^^15^ The phenotypic presentation of individuals with rare variant genotypes is complex and involves other psychiatric conditions^16^^,^^17^ as well as cognitive impairment.^8^^,^^18^, ^19^, ^20^

Whilst the number of rare variants reported to be associated with autism continues to grow,^4^, ^5^, ^6^^,^^21^ understanding of the phenotypic presentation is lacking. The extent to which different genotypes are associated with different aspects of the autism phenotype remains unclear, and predicting specific profiles in individuals with autism can be complicated by the frequently multimorbid presentation as well as incomplete penetrance and pleiotropy.^10^^,^^11^^,^^16^^,^^18^ These issues currently make using genetic information in the clinical setting highly challenging.^22^ Although several studies have described the autism phenotype in individuals with rare variant genotypes,^23^, ^24^, ^25^ there is a sparsity of studies examining the extent to which genetic heterogeneity underpins phenotypic heterogeneity.^24^^,^^26^^,^^27^

Several studies have described the phenotypic profiles associated with specific rare variant genotypes, but these reports are limited by small sample sizes and the small number of variants investigated, e.g.,.^23^^,^^25^, ^26^, ^27^, ^28^, ^29^ Only a few studies to date^24^^,^^25^^,^^28^^,^^30^ have examined whether autism profiles differ between individuals with rare variant genotypes and those with idiopathic autism (i.e., autism of unknown genetic origin). Investigating these issues will elucidate whether individuals with autism with rare variant genotypes would benefit from different types of support compared to those with idiopathic autism and inform care strategies. Previous work^24^ recently compared individuals with deletions and duplications of 16p11.2 and 22q11.2 using the semi-structured research diagnostic Autism Diagnostic Interview (ADI-R).^31^ This work found subtle differences in autism profiles between the genotypes but much more substantial phenotypic variation within each genotype.^24^ Furthermore, over half (54%) of those who did not meet the diagnostic criteria for autism still exhibited clinically significant symptoms.^24^ These findings, however, focussed on only two genomic loci, and the extent to which the findings apply to a broader range of ND-CNVs as well as SGVs remains to be determined.

The Social Communication Questionnaire (SCQ) is a widely utilised tool to assess autism-related presentations in clinical practice and research.^32^, ^33^, ^34^ It was developed based on the ADI-R^31^ and has been shown to have high specificity (80%)^35^ and an acceptable diagnostic accuracy (area under the curve (AUC) = 0.88),^34^ making it a cost-effective option for autism screening in large cohorts. A score of ≥22 on the SCQ indicates a need for a clinical evaluation of autism.^32^, ^33^, ^34^ However, it is important to note that the SCQ does not provide a definitive diagnosis of autism. In this work, we will refer to individuals who screened positive for autism on the SCQ as having “indicative autism” and to scores on the three sub-domains (social, communication, and repetitive behaviour) as “autism subdomain scores”.

Our study aims to investigate differences in indicative autism scores across a range of rare variant genotypes. To address this question, we have assembled a sample of 1314 young people with one of 30 rare variants, including 15 ND-CNVs (comprising losses and gains of various-sized segments across eight different chromosomes), as well as 15 SGVs. These 30 rare variant genotypes were selected because of a strong documented association with autism.^4^, ^5^, ^6^, ^7^ SCQ scores of these young people were compared to those of similar-aged neurotypical individuals without a known genetic condition (controls; n = 460) as well as with individuals with idiopathic autism (n = 480). We aimed to answer the following research questions:

• To what extent do autism prevalence and subdomain scores differ across rare variant genotypes, and what proportion of variation is explained by genotype?
• Do the autism subdomain scores of individuals with rare variant genotypes differ from those with idiopathic autism?

## Methods

### Study cohorts

#### Individuals with rare variant genotypes

1314 individuals with either one of 15 ND-CNVs (n = 1005, mean age 9.2 ± 3.5 years, 60% male) or 15 SGVs (n = 309, mean age 8.3 ± 4.0 years, 50% male) were identified across four different sites (Table 1). The ND-CNV cohort consisted of 1005 young people with one of 15 ND-CNV across 8 chromosomal regions (9.2 ± 3.5 years, 60% male), which prior studies have associated with increased autism liability^26^^,^^30^^,^^36^^,^^37^ (Table 2). The SGVs cohort comprised 309 individuals (8.3 ± 4.0 years, 50% males) with variants in one of 15 genes. These variants included single nucleotide variants (de novo loss-of-function (LoF) variants, nonsense/frameshift variants) and deletions or duplications within genes known to be associated with autism^38^, ^39^, ^40^ (Table 2).

**Table 1:** Demographic information and prevalence of indicative autism and being non-verbal across the cohorts.

Cohorts	Sample size	Sex		Age		Indicative autism^a^		Non-verbal	
	n	n	% (male)	Mean	SD	n	%	n	%
ND-CNV cohort									
Cardiff University cohort	493	310	63	9.5	3.1	144	29	28	6
Simons Searchlight cohort	364	209	57	8.4	3.7	87	24	39	11
UCLA^b^ cohort	79	40	51	11.6	3.5	3	4	1	1
The 3q29 project cohort	69	40	58	9.1	3.7	18	26	12	17
Total	1005	599	60	9.2	3.5	252	25	80	8
SGV cohort									
Simons Searchlight cohort	309	153	50	8.3	4.0	163	53	176	57
Control cohort									
Cardiff University cohort	208	112	54	10.4	2.8	8	4	1	0
Simons Searchlight cohort	166	83	50	10.4	3.7	0	0	0	0
UCLA cohort	45	26	58	12.0	3.3	2	4	3	7
The 3q29 project cohort	41	22	54	10.4	4.2	0	0	0	0
Total	460	243	53	10.6	3.4	10	2	4	1
Idiopathic autism cohort^c^									
SPARK cohort	480	389	81	8.6	3.2	480	100	59	12

ND-CNVs = neurodevelopmental risk copy number variants. SGVs = single gene variants. SPARK = Simons Foundation Powering Autism Research for Knowledge.

a Based on a cut-off score of ≥22 on the Social Communication Questionnaire (SCQ).

b UCLA University of California at Los Angeles.

c Idiopathic autism = autism with unknown genetic origin.

**Table 2:** Demographic information and prevalence of indicative autism and being non-verbal for the rare variant genotypes.

Rare variant genotypes	Sample size n	Sex		Age		Indicative autism^a^		Non-verbal	
		n	% male	Mean	SD	n	%	n	%
ND-CNVs^b^									
1q21.1 deletion	47	30	64	8.2	3.5	14	30	2	4.3
1q21.1 duplication	67	39	58	8.7	3.4	32	48	7	10.4
1q21.1 TAR duplication	14	8	57	8.5	2.7	3	21	2	14.3
NRXN1 deletion	19	16	84	8.4	2.7	8	42	2	10.5
3q29 deletion	61	39	64	9.2	3.8	14	23	10	16.4
3q29 duplication	13	5	38	8.5	3.3	6	46	3	23.3
Kleefstra syndrome	13	5	38	11.6	3.9	7	54	1	7.7
15q13.3 deletion	27	21	78	9.4	3.8	11	41	1	3.7
15q13.3 duplication	23	15	65	9.4	3.2	8	35	1	4.3
15q11.2 deletion	42	32	76	9.2	3.1	16	38	3	7.1
16p11.2 deletion	254	146	57	8.8	3.6	48	19	26	10.2
16p11.2 distal deletion	24	14	58	9.4	3.1	9	38	5	20.8
16p11.2 duplication	125	76	61	10.2	3.6	39	31	8	6.4
22q11.2 deletion	212	114	54	10.1	3.1	22	10	5	2.4
22q11.2 duplication	64	39	61	10.1	3.4	14	22	4	6.2
Total	1005	599	60	9.2	3.5	252	25	80	8
SGVs^c^									
*ADNP*	13	8	62	9.5	4.2	11	85	7	53.8
*ASXL3*	20	10	50	8.9	3.9	14	70	17	85
*CTNNB1*	16	6	38	9.1	4.7	6	38	5	31.2
*DYRK1A*	13	9	69	8.5	4.8	11	85	8	61.5
*GRIN2B*	27	18	67	8.6	4.0	15	56	12	44.4
*HIVEP2*	11	8	73	7.3	2.8	3	27	4	36.4
*HNRNPH2*	11	1	9	10.5	5.3	6	55	8	72.7
*MED13L*	11	10	91	7.9	4.3	6	55	8	72.7
*PACS1*	17	6	35	7.6	4.1	7	41	7	41.2
*PPP2R5D*	33	12	36	7.4	3.6	14	42	15	45.5
*SCN2A*	52	23	44	8.1	4.3	31	60	38	73.1
*SETBP1*	10	7	70	7.5	3.5	2	20	7	70
*SLC6A1*	27	15	56	8.1	3.8	7	26	6	22.2
*STXBP1*	26	9	35	8.6	4.1	19	73	23	88.5
*SYNGAP1*	22	11	50	7.6	3.3	11	50	11	50.0
Total	309	153	50	8.3	4.0	163	53	176	57

ND-CNVs = neurodevelopmental risk-copy number variations.

SGVs = single gene variants.

a Based on a cut-off score of ≥22 on the Social Communication Questionnaire (SCQ).

b ND-CNV regions included in the study are: 1q21.1 (critical region 145.3–147.3), 2p16.3 NRXN1 (critical region 50.1–51.2), 3q29 (critical region 192.5–198.0), 9q34 (critical region 140.5–140.7), 15q11.2 (critical region 22.8–23.1), 15q13.3 (critical region 31.10–32.4), 16p11.2 (critical region 28.8–30.2), and 22q11.2 (critical region 19.0–21.5).

c SGVs included in the study are: *ADNP* variants (chromosome band 20q13.13); *ASXL3* variants (chromosome band 18q12.1); *CTNNB1* variants (chromosome band 3p22.1); *DYRK1A* variants (chromosome band 21q22.13); *GRIN2B* variants (chromosome band 12p13.1); *HIVEP2* variants (chromosome band 6q24.2); *HNRNPH2* variants (chromosome band Xq22.1); *MED13L* variants (chromosome band 12q24.21); *PACS1* variants (chromosome band 12q24.21); *PPP2R5D* variants (chromosome band 6p21.1); *SCN2A* variants (chromosome band 2q24.3); 13 *SETBP1* variants (chromosome band 18q12.3); *SLC6A1* variants (chromosome band 3p25.3); *STXBP1* variants (chromosome band 9q34.11); and *SYNGAP1* variants (chromosome band 6p21.32).

At Cardiff University, data was collected as part of the Cardiff University ECHO (Experiences of People With Copy Number Variants) https://www.cardiff.ac.uk/centre-neuropsychiatric-genetics-genomics/research/themes/developmental-psychiatry/copy-number-variant-research-group and the IMAGINE-ID Intellectual Disability and Mental Health: Assessing Genomic Impact on Neurodevelopment https://www.ucl.ac.uk/child-health/research/population-policy-and-practice-research-and-teaching-department/cenb-clinical-29 studies (n = 493). These two studies contributed data on 15 ND-CNVs (Supplementary Table S1).

The University of California Los Angeles (UCLA) contributed data on individuals with 22q11.2 deletion or duplication (n = 79) (https://www.semel.ucla.edu/bearden-lab), and the 3q29 Project at Rutgers University on individuals with 3q29 deletion or duplication (n = 69) (https://sites.rutgers.edu/mulle/). Finally, Simons Searchlight project contributed data on individuals with 15 SGVs (n = 309) and 5 CNVs ((1q21 deletion and duplication (n = 64), 16p11.2 deletion (n = 207), and 16p11.2 duplication (n = 93)) (https://www.sfari.org/resource/simons-searchlight/) (Supplementary Table S1)). SGVs included single nucleotide variants (de novo loss-of-function (LoF) variants, nonsense/frameshift variants) and deletions or duplications within genes known to be associated with autism.

#### Individuals with idiopathic autism

The idiopathic autism cohort was recruited through the Simons Foundation Powering Autism Research for Knowledge (SPARK) project (https://www.sfari.org/resource/spark/). The SPARK registry comprises a cohort of approximately 99,000 children and adults with a clinical diagnosis of autism.^41^ For the current paper, we first excluded adults and individuals with incomplete/missing items on the SCQ and incomplete IQ data. Finally, we excluded all individuals with known rare pathogenic CNVs/SGVs. We did not include this latter excluded group in our rare variant cohort because recruitment differences could have biased our findings. The idiopathic autism cohort included in this study thus comprised 480 young people (8.6 ± 3.2 years (81% male)) who were comparable to the rare variant genotypes cohort in age and sex (Table 1).

#### Neurotypical control participants

All sites that contributed individuals with rare variant genotypes also recruited neurotypical participants, resulting in a combined sample of 460 controls (10.6 ± 3.4 years, 53% male) (Table 1). These were either siblings of individuals with rare variant genotypes (familial controls, n = 329, age (8.3 ± 4.0 years, 51% males)) or unrelated children (community-based controls, n = 138, age (11.6 ± 3.5 years, 56% males)).^26^^,^^37^^,^^38^^,^^42^ The absence of neurodevelopmental risk variants in the siblings was confirmed through medical records and/or genotyping in the laboratories of the contributing sites. This information was, however, not available for the community-based controls. Both groups were comparable to the rare variant genotype cohorts in age and sex and were assessed with the same measures. Indicative autism prevalence (2% in familial controls and 1% in community-based controls) was comparable between the two control groups (OR = 1.6, CI = 0.36–8.1, p = 0.736 (mixed effects logistic regression)) as were autism subdomain scores (familial control mean total SCQ score 3.7 ± 4.1, community-based controls mean total SCQ score 4.3 ± 5.6, p = 0.910). We, therefore, combined these two groups into one control cohort.

### Phenotype assessments

#### Indicative autism prevalence and subdomain scores

In this study, all primary caregivers of participants—including those with rare variant genotypes, idiopathic autism, and controls—were requested to complete the SCQ to screen for autism. The SCQ consists of 40 yes/no questions that are scored based on the presence or absence of autism features.^32^ The social subdomain score assesses the child's ability to interact socially, including their facial expressions, play, and ability to form friendships. This subdomain contributes 20 points to the total score. The communication subdomain score evaluates the child's use of language and nonverbal communication and contributes 11 points to the total score. The repetitive behaviour subdomain score assesses whether the child has restricted interests and engages in repetitive behaviour and contributes 8 points to the total score. These scores are commonly referred to as “autism subdomain scores”.^32^

The SCQ distinguishes between verbal and nonverbal children, asking, “Is she/he now able to talk using short phrases or sentences?” There are 7 questions that are only applicable to verbal children. Therefore, the total SCQ score ranges from 0 to 39 for verbal children, whereas for nonverbal children, it ranges from 0 to 32. Numbers and percentages of non-verbal participants were: ND-CNV (n = 80 (8%)); SGV (n = 176 (57%)); idiopathic autism (n = 48 (10%)). We used a previously published method^32^ to adjust the total and communication subdomain scores for non-verbal participants to take into account the 7 missing items. We also performed a sensitivity analysis to assess how excluding nonverbal participants affected our findings.

A higher SCQ score usually indicates an increased likelihood of autism. We utilised the established cut-off of ≥22 as a positive indicator for autism screening.^32^ This threshold effectively predicts autism, particularly in clinical populations,^43^^,^^44^ and serves as a clinical reference for further evaluation using the ADI-R. It is worth noting that prior research frequently used a cut-off of ≥15 to indicate potential autism spectrum traits.^26^^,^^32^ The SCQ has two versions: lifetime and current.^45^ The lifetime version queries observed behaviour across the child's lifespan, whereas the current version focuses on symptoms during the last three months. Although the two versions are functionally and psychometrically similar, the current version is used mainly for follow-up and evaluation of any interventions.^34^ The lifetime version was administered in three cohorts (Cardiff, Simons Searchlight, and SPARK; individuals with rare variant genotypes n = 857, controls n = 374, idiopathic autism n = 469) and the current version in two (the 3q29 Project and UCLA; individuals with rare variants n = 148, controls n = 86). The two versions were combined in analyses, and we conducted a sensitivity analysis to evaluate whether the findings were influenced by which version was used.

#### Cognitive profile

IQ assessments were conducted for individuals with ND-CNVs and controls from Cardiff University, as well as the Simons Searchlight and UCLA cohorts, but not the 3q29 Project cohort. IQ data was not available for individuals with SGVs. The sites that contributed IQ data used either the Wechsler Abbreviated Scale of Intelligence (WASI)^46^ or the Wechsler Intelligence Scale for Children (WISC-V).^47^ Full-scale IQ (FSIQ), Performance IQ (PIQ), and Verbal IQ (VIQ) scores were derived from these scales.^48^ The SPARK cohort obtained data on IQ for children with idiopathic autism from health records.

#### Other conditions associated with autism and medication use

Information on other conditions associated with autism was available for a subset of individuals with rare variant genotypes. These included attention deficit hyperactivity disorder (ADHD), seizures, oppositional defiant disorder, conduct disorder, depression, and tic disorder, as well as sleep problems as derived from semi-structured psychiatric interviews (Child & Adolescent Psychiatric Assessment (CAPA) at Cardiff University^26^) or clinical notes, and/or other primary carer-reported measures at the other sites^36^, ^37^, ^38^^,^^42^ (Supplementary Table S2 for details).

Data on medication use (antipsychotics, anticonvulsants, mood stabilisers or ADHD treatment) (Supplementary Table S2), socioeconomic status (income and education), and ethnicity were obtained for those with ND-CNVs, SGVs, idiopathic autism and controls. Medication use, socioeconomic status, and ethnicity were included as covariates in sensitivity analyses.

### **Statistical analysis**

#### Indicative autism prevalence across individuals with rare variant genotypes

Mixed effects logistic regression models^49^ were used to determine whether the prevalence of indicative autism (outcome) differed by rare variant genotype (predictor). These comparisons were conducted between 1) individuals with rare variant genotypes versus controls; 2) individuals with rare variant genotypes (either ND-CNVs or SGVs) versus controls; 3) individuals with ND-CNVs versus those with SGVs; and 4) each of the 30 rare variant genotypes versus controls.

In each model, age and sex (self-reported by study participants or, where applicable, their caregiver) were included as fixed effects. In line with previous studies, study site (Europe versus United States) and family status (accounting for that fact that a subset of participants with rare variant genotypes and controls came from the same family) were included as random effects.^24^^,^^26^^,^^50^ The outcome of each model represented the odds of having indicative autism in each group compared to the other group. For comparison of the 30 rare variant genotypes versus controls, post hoc comparisons were subsequently conducted to determine group contrasts. All p-values were corrected for multiple comparisons using Tukey's Honest Significant Difference (HSD) adjustment.^51^

#### Autism total and subdomain scores across individuals with rare variant genotypes

We used mixed-effects linear regression models^49^ to compare autism total and subdomain scores (the outcomes) first between individuals with ND-CNVs versus those with SGVs and then across the 30 different variant genotypes. We included study site and family status as random effects and age and sex as fixed effects. Post-hoc contrasts were conducted to determine between-group contrast estimates with Tukey's HSD adjustment of p-values.^51^

#### Variation in autism total and subdomain scores explained by rare variant genotypes

To examine the variation in autism total and domain scores between rare variant genotypes, we conducted analysis of covariance (ANCOVA). In each ANCOVA model, the outcome (SCQ total and each of the three subdomain scores) was predicted by rare variant genotype with age, sex, and study site as covariates. We determined the proportion of variance in these scores that is attributable to rare variant genotype (between-genotype variation) using eta-square (*η*^2^),^52^ as in previous studies.^24^ The within-genotype variation was then calculated as the variation remaining after accounting for between-genotype variation and variation attributed to covariates (age, sex, and study site) (the three sources of variation summing to 100).

#### Autism total and subdomain scores in individuals with rare variant genotypes who screen positive for autism and individuals with idiopathic autism

We conducted comparisons between individuals with rare variant genotypes who tested positive for autism (SCQ score ≥ 22): ND-CNVs with indicative autism (ND-CNVs_ia; n = 238) and SGVs with indicative autism (SGVs_ia; n = 120) compared to individuals with idiopathic autism (n = 480). Mixed-effects linear regression models were conducted, as explained above. We also assessed whether specific rare variant genotypes yield different total and subdomain scores when compared to individuals with idiopathic autism. This analysis included rare variant genotype groups that had a minimum of 10 participants who scored positively for autism, specifically 14 rare variant genotypes (9 ND-CNVs_ia; n = 199 and 5 SGVs_ia; n = 70). Post-hoc contrasts were conducted to determine between-group contrast estimates with Tukey's HSD adjustment of p-values.^51^

### Sensitivity analyses

#### Ethnic background, socioeconomic status, other conditions associated with autism and medication use

Sensitivity analyses were performed by including ethnic background, socioeconomic status, relevant conditions, and medication use (Supplementary Table S2) as covariates in the models outlined earlier to determine if these factors influenced our findings.

#### Inclusion of IQ as a covariate

We accounted for FSIQ, VIQ, and PIQ individually in each model mentioned above. Since not all sites assessed IQ, this analysis was confined to a subsample of 701 individuals with rare variant genotypes (53.3%), 320 controls (69.6%), and 480 individuals with idiopathic autism (100%).

#### Analysis of data using the lifetime SCQ version only

The Cardiff, Simons Searchlight, and SPARK cohorts used the SCQ lifetime version (individuals with rare variants n = 1166, controls n = 374, idiopathic autism n = 469), while the current version was used by the 3q29 project (individuals with rare variants n = 69, controls n = 41) and UCLA (individuals with rare variants n = 79, controls n = 45). To evaluate if the inclusion of both versions in our analysis impacted the findings, we ran our models excluding data collected with the SCQ current version (administered by the 3q29 project and UCLA). This analysis was based on 1166 individuals with rare variant genotypes (84.9% of the total sample) and 374 controls (81.3% of the total sample).

#### Exclusion of nonverbal individuals

The ND-CNV cohort included 80 (8%), the SGV cohort 176 (57%), and the idiopathic autism cohort 48 (10%) nonverbal individuals. To evaluate the impact of including nonverbal participants, we reran the models, excluding those who were nonverbal. This analysis was based on n = 925 individuals with ND-CNVs (92% of the total cohort with ND-CNVs), n = 133 (43%) individuals with SGVs, and n = 421 (90%) individuals with idiopathic autism.

All analyses were performed using R version 4.4.1. An alpha level of 0.05 was used to calculate multiple testing thresholds.

### Ethics

All procedures involving human subjects/patients were approved by the appropriate local ethics committees or institutional review boards.^26^^,^^36^, ^37^, ^38^^,^^41^^,^^42^^,^^53^^,^^54^ Recruitment and assessment protocols for the ECHO study were approved by The South-East Wales Research Ethics Committee (09/WSE04/22),^53^ while those of the IMAGINE-ID^26^ were approved by the NHS London Queen Square research ethics committee (14/LO/1069). The 3q29 project was approved by Emory University's Institutional Review Board (IRB00064133) and Rutgers University's Institutional Review Board (Pro2021001708). The UCLA Institutional Review Board approved the UCLA Study.^54^ Further details on Simon's Searchlight and the SPARK cohorts' ethical approval can be found at the website https://www.sfari.org/resource/simons-searchlight/.^38^^,^^41^

Before recruitment, written consent or assent was obtained from each participant and, where applicable, their caregiver. The presence of rare variant genotypes was confirmed through microarray analysis at the laboratories of participating clinical research sites and/or collected via medical records.

### Role of funders

None of the funders had any role in study design, data collection, data analyses, interpretation, or the writing of this manuscript.

## Results

### Indicative autism prevalence across individuals with rare variant genotypes

Individuals with rare variant genotypes had a higher prevalence of indicative autism than controls (32% vs 2%; OR = 43.1, CI = 6.6–282.2, p < 0.001 (mixed effects logistic regression)). Those with SGVs were more likely to have indicative autism compared to those with ND-CNVs (53% vs 25%; OR = 4.00, CI = 2.2–7.3, p = 0.002 (mixed effects logistic regression)). We also calculated these prevalences using a cut-off of ≥15 on the SCQ to allow for comparison with other studies that used the same threshold. As anticipated, the rates increase with the less strict cut-off; however, the pattern remains consistent (Supplementary Table S3).

**Fig. 1:**
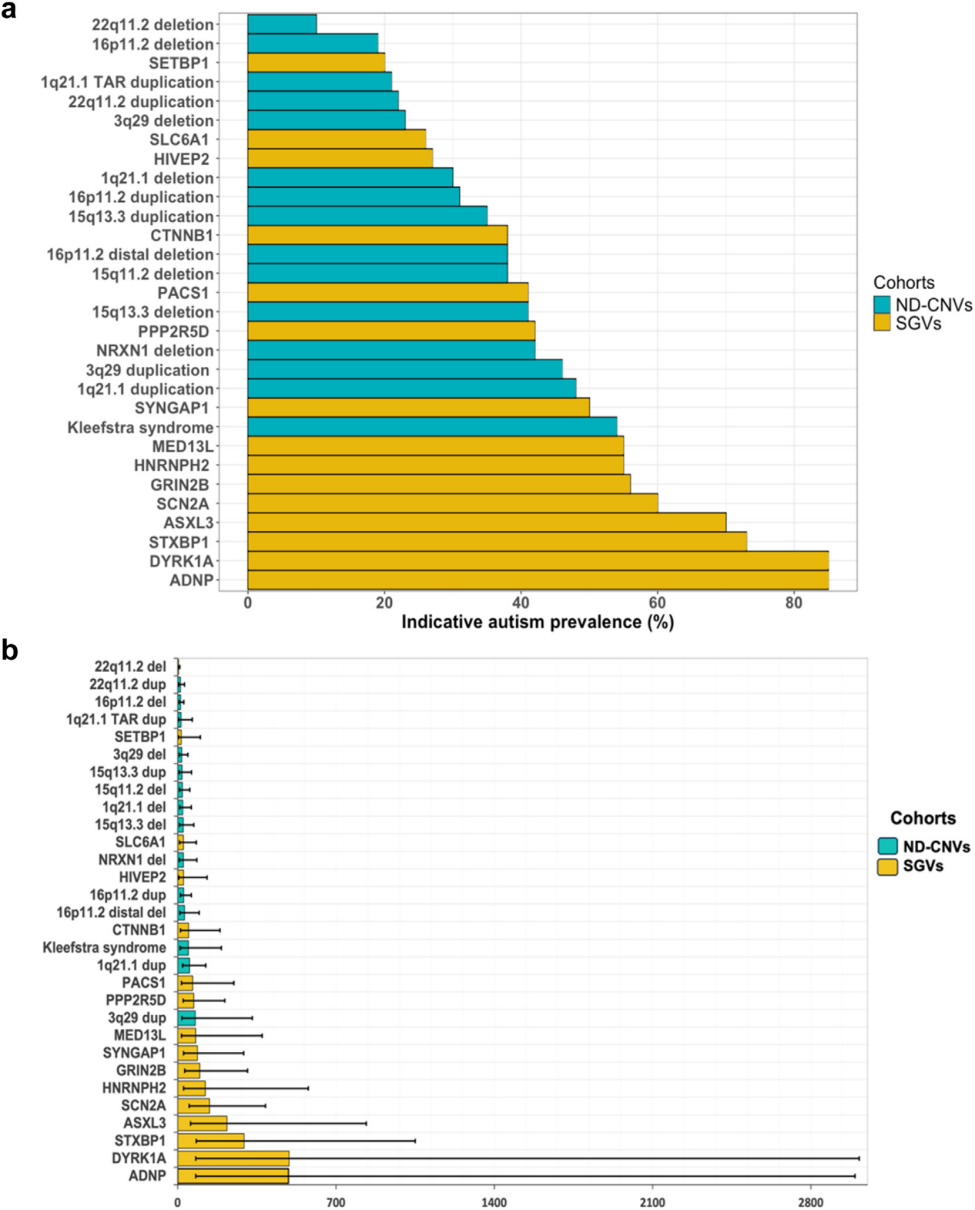
**Prevalence and odds ratios of indicative autism in individuals with rare variants genotypes. a. Prevalence of indicative autism in individuals with rare variant genotypes**. The figure illustrates the percentages of individuals who passed the autism cut-off of SCQ ≥ 22 (indicative autism) in the 30 rare variant genotypes. ND-CNVs = neurodevelopmental risk-copy number variants. SGVs = single gene variants. **b. Odds ratios of having indicative autism in individuals with rare variant genotypes compared to controls**. The figure illustrates the odds of having indicative autism in individuals with rare variant genotypes compared to controls. The ORs were derived from mixed-effects logistic regression models. All p-values were significant after correction for multiple testing except for the comparisons of 1q21 TAR duplication and SETBP1 with the control sample. ND-CNVs = neurodevelopmental risk-copy number variants. SGVs = single gene variants.

The prevalence of indicative autism was variable across the 30 rare variant genotypes, being highest in individuals with *ADNP* variants (85%, OR in comparison to controls = 489.8, CI = 80.08–2995.41, p < 0.001 (mixed effects logistic regression)) and lowest in individuals with 22q11.2 deletion (10%, OR in comparison to controls = 4.2, CI = 1.94–9.02, p = 0.042 (mixed effects logistic regression)) (Fig. 1a, Supplementary Table S4). However, it should be noted that wide and overlapping confidence intervals existed for these estimates (Fig. 1b). Comparing the prevalence of indicative autism across the 30 rare variant genotypes revealed several significant differences (34 out of 435), mostly showing lower prevalence in those with 22q11.2 deletions or duplications or 16p11.2 deletions or duplications compared to those with SGVs (Supplementary Table S5).

### Autism total and subdomain scores across individuals with rare variant genotypes

**Fig. 2:**
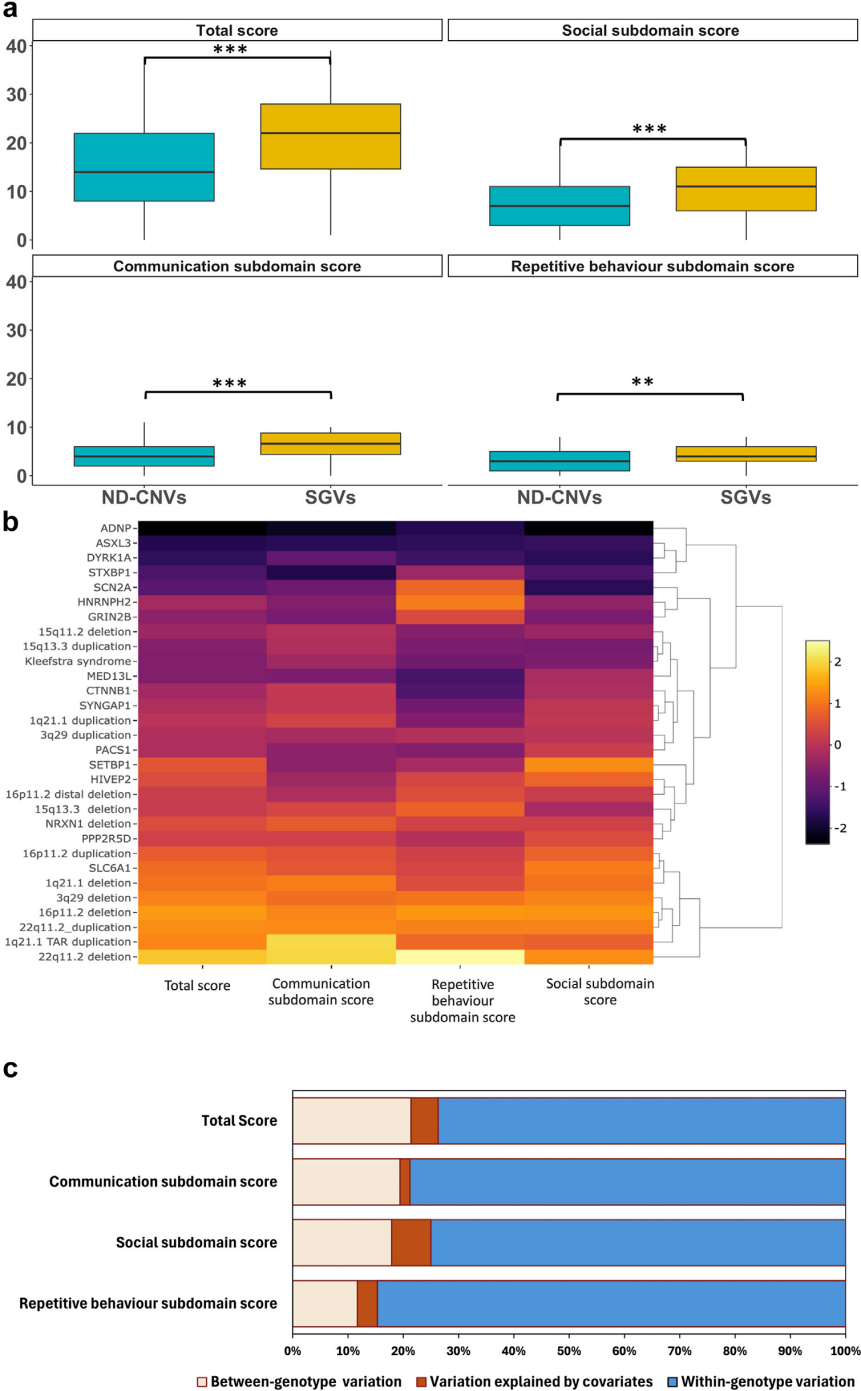
**Autism subdomain scores across individuals with rare variant genotypes. a. Autism total and subdomain scores in individuals with ND-CNVs compared to those with SGVs**. This figure shows the mean total and subdomain scores in individuals with SGVs compared to those with ND-CNVs. ND-CNVs = neurodevelopmental risk-copy number variants. SGVs = single gene variants. ∗∗p < 0.01, ∗∗∗p < 0.001 (mixed effects linear regression). **b. Autism total and subdomain scores variation across individuals with rare variant genotypes**. This heatmap plot was generated by standardising the total and subdomain scores of each rare variant genotype (compared to the mean score of all variants) using z scores. Darker colours indicate relatively higher scores in a domain (more autism symptoms are endorsed—greater impairment). It shows subdomain-specific patterns across the 30 rare variant genotypes. **c. Variation in autism total and subdomain scores explained by rare variant genotype**. The figure illustrates the between-group and within-group variation in autism domain scores. The within-group variation is considerably larger than the between-group variation. Between-genotype variation: variation explained by rare variant genotype, as measured *η*^2^ (eta-squared). Variation explained by covariates: age, sex, and study site. Within-genotype variation: Variation remaining within each variant genotype after considering the between-genotype variation and variation explained by other covariates (see Methods for further explanation).

Individuals with SGVs had higher SCQ total scores than those with ND-CNVs (group contrast estimates = 6.9, CI = 4.6–9.3, p < 0.001 (mixed effects linear regression)), indicating greater social disability in these groups. They also showed greater impairment in the social (group contrast estimates = 3.9, CI = 2.6–5.3, p < 0.001 (mixed effects linear regression)), repetitive behaviour (group contrast estimates = 0.9, CI = 0.3–1.4, p = 0.009 (mixed effects linear regression)) and communication subdomains (group contrast estimates = 1.9, CI = 1.3–2.7, p < 0.001 (mixed effects linear regression)) (Fig. 2a, Supplementary Table S6).

Comparisons of total and subdomain scores across rare variant genotypes showed that individuals with 22q11.2 deletion generally showed the least impairment, whereas the opposite was true for those with *ADNP* variants (Fig. 2b, Supplementary Table S7). Subdomain-specific patterns were also present; for example, individuals with *STXBP1* were relatively impaired in the communication subdomain, those with *SCN2A* variants in the social, and those with *MED13L* variants in the repetitive behaviour subdomain. On the other hand, individuals with 1q21 TAR duplication showed relative strength in the communication subdomain, those with *HNRNPH2* variants in the repetitive behaviour, and those with *SETBP1* variants in the social subdomain (Fig. 2b, Supplementary Table S7).

### Variation in autism total and subdomain scores explained by rare variant genotypes

Rare variant genotypes contributed to variation in autism total as well as the three subdomain scores. However, the proportion of variance explained was relatively low (<22%). Indeed, the variation in autism total score within rare variant genotypes was substantially greater than between-genotype (*η*^2^ = 73.7% vs *η*^2^ = 21.4%, p < 0.0001 (analysis of covariance)) .Similarly, the within-genotype variation for the three subdomain scores (*η*^2^ = 75% for social subdomain, *η*^2^ = 78.8% for communication subdomain, and *η*^2^ = 84.6% for repetitive behaviour subdomain) was considerably greater than between-genotype variation (*η*^2^ = 17.9% for social subdomain (p < 0.0001 (analysis of covariance)), *η*^2^ = 19.4% for communication subdomain (p < 0.0001 (analysis of covariance)) and, *η*^2^ = 11.8% for repetitive behaviour subdomain (p < 0.0001 (analysis of covariance))) (Fig. 2c, Supplementary Table S8).

### Autism total and subdomain scores in individuals with rare variant genotypes who screen positive for autism and individuals with idiopathic autism

**Fig. 3:**
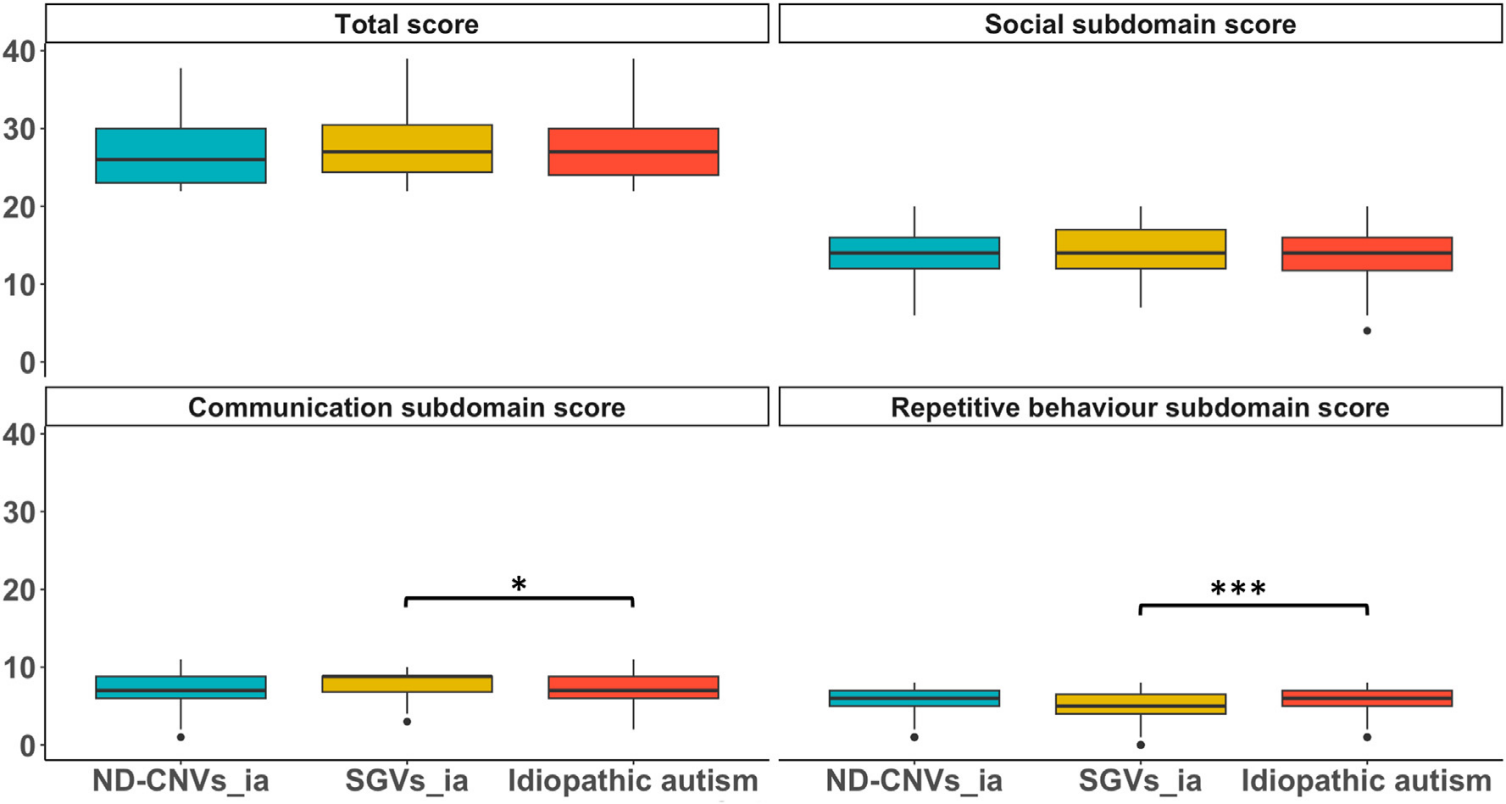
**Autism subdomain scores in individuals with rare variant genotypes compared to those with idiopathic autism**. This figure shows the mean total and subdomain scores in individuals with ND-CNVs and SGVs compared to those with idiopathic autism. ND-CNVs_ia: individuals with ND-CNVs who passed the cut-off of ≥22 for autism on the SCQ. SGVs_ia individuals with SGVs who passed the cut-off of ≥22 for autism on the SCQ. ∗p < 0.05, ∗∗∗p < 0.001 (mixed effects linear regression).

Individuals with ND-CNVs_ia (n = 252) showed similar total and subdomain scores to those with idiopathic autism (Fig. 3, Supplementary Table S9). Individuals with SGVs_ia (n = 163) also showed comparable total score to those with idiopathic autism. However, they showed greater impairment in the communication subdomain (group contrast estimates = 0.46, CI = 0.17–0.75, p = 0.012 (mixed effects linear regression)) and less impairment in the repetitive behaviour subdomain (group contrast estimates = −1.18, CI = −1.51 to −0.88, p < 0.001 (mixed effects linear regression)) (Fig. 3, Supplementary Table S9).

Post hoc analysis comparing individuals with rare variants who scored positive for autism with those with idiopathic autism showed several differences (Supplementary Table S10). Individuals with *SCN2A* variants showed more social impairment (group contrast estimates = 2.71, CI = 1.59–3.83, p < 0.001 (mixed effects linear regression)) but less impairment in the repetitive behaviour subdomain (group contrast estimates = −2.23, CI = −2.85 to −1.61, p < 0.001 (mixed effects linear regression)). Those with *GRIN2B* variants and 22q11.2 deletion showed less impairment in the repetitive behaviour subdomain (group contrast estimates = −2.43, CI = −3.3 to 1.56, p < 0.001; group contrast estimates = −1.37, CI = −2.09 to −0.64, p = 0.028 (mixed effects linear regression)) respectively. Furthermore, individuals with 22q11.2 deletion showed less impairment in the communication subdomain compared to those with idiopathic autism (group contrast estimates = −1.48, CI = −2.18 to −0.78, p = 0.006 (mixed effects linear regression)) (Supplementary Table S10).

### Sensitivity analysis

#### Ethnic background, socioeconomic status, other conditions associated with autism and medication

All findings remained consistent after adjusting for ethnicity, socioeconomic status, medication usage, and other conditions (Supplementary Table S11).

#### Inclusion of IQ as a covariate

The inclusion of FSIQ, Verbal IQ, or PIQ as a covariate in the analysis did not change the findings comparing between ND-CNVs and controls or across ND-CNVs (Supplementary Table S12).

#### Analysis of data using the lifetime SCQ version only

The exclusion of individuals assessed with the SCQ current rather than the lifetime version also did not impact the results (Supplementary Table S13).

#### Exclusion of nonverbal individuals

After excluding nonverbal individuals, the results stayed consistent, except for the loss of difference in the repetitive subdomain between individuals with SGVs_ia and those with idiopathic autism (Supplementary Table S14).

## Discussion

Elucidation of the contribution of different rare variant genotypes to variability in autism phenotype requires sufficient numbers of participants across a range of genotypes assessed with the same measures.^22^^,^^24^^,^^26^^,^^27^ Through international collaboration, this study allowed the comparison of autism subdomain scores in a cohort of 2254 participants, including 1314 young people with one of 30 distinct ND-CNVs or SGVs that have previously been robustly associated with autism, alongside 480 individuals with idiopathic autism and 460 neurotypical controls of comparable age.

The presence of a rare variant genotype was associated with a 43-fold increased likelihood of indicative autism compared to controls. Our findings highlighted differences in indicative autism prevalence and subdomain profiles between rare variant genotypes. Specifically, individuals with SGVs had a higher prevalence of indicative autism and were more impaired in the social, communication and repetitive behaviour subdomains compared to those with ND-CNVs. Furthermore, individuals with SGVs who screened positive for autism (SGVs_ia) showed a higher burden of communication difficulties and a lower burden of repetitive behaviour compared to those with idiopathic autism. Genotype explained between 11.7% and 21.4% of the variation in autism total and subdomain scores, whereas the variation within the genotypes was substantially larger (between 73.7% and 84.6%). Although our findings indicate that individual rare variant genotypes vary in prevalence and subdomain scores, overall, there is greater evidence of convergence of phenotype across genotypes rather than divergence. It is important to emphasise, however, that considerable heterogeneity existed within the genotypes, highlighting that these rare variants are not fully penetrant for autism and that other factors will also be important for the prediction of phenotypic variability.

Many ND-CNVs and SGVs have now been implicated in autism liability.^4^, ^5^, ^6^^,^^17^ However, a limited number of studies to date have compared autism phenotypes across a range of variants.^23^^,^^25^, ^26^, ^27^, ^28^, ^29^ We observed considerable variation in indicative autism prevalence across ND-CNVs and SGVs, with the presence of SGVs being associated with a higher prevalence of indicative autism than ND-CNVs. Although there was considerable variation across SGV genotypes, 9 of the 10 most penetrant variants were SGVs (Fig. 2b). This might be expected since the specific SGVs we studied were selected based on their strong association with autism.^39^ The lowest prevalence of autism was found in individuals with 22q11.2 deletion (10%, OR = 4.19; CI = 1.94–9.02, p = 0.042 (mixed effects logistic regression)), consistent with reports comparing autism prevalence in individuals with 22q11.2 to those with other ND-CNVs.^23^^,^^24^^,^^55^ These findings are similarly consistent with previous research that has reported that autism penetrance varies across genetic aetiologies.^23^^,^^24^^,^^48^^,^^56^^,^^57^

Previous studies have reported qualitative and quantitative differences in autism phenotype in individuals with rare variant genotypes.^24^^,^^26^, ^27^, ^28^, ^29^ Our findings indicate that individuals with SGVs were more impaired in terms of having higher autism domain scores than those with ND-CNVs. SGVs are known to impact cognitive function, and our findings are consistent with recent research reporting individuals with SGVs are more likely to exhibit severe autism symptoms and suggest higher comorbidity with intellectual disability compared to those with ND-CNVs.^21^^,^^58^^,^^59^ Our findings stress the need for further detailed comparative studies encompassing individuals with both SGVs and ND-CNVs. This is a research area of growing importance as SGVs are increasingly diagnosed in clinical settings.

We observed distinct patterns across the 30 rare variant genotypes, as illustrated in Fig. 2b. Those with 22q11.2 deletion were generally least impaired across total and subdomain scores, while those with *ADNP* variants tended to be most impaired. Subdomain-specific patterns were also found, indicating relative strengths for those with 1q21 TAR duplication in the communication subdomain, those with 16p11.2 deletion in the social subdomain and those with 16p11.2 deletion and *HNRNPH2* variation in the repetitive behaviour subdomain. Relative weaknesses were found in the communication subdomain for those with *STXBP1* variation, the social subdomain for those with SC2NA variation, and the repetitive behaviour subdomain for those with 15q11.2 deletion and *ASXL3* variation. These findings underscore the importance of comprehensive phenotypic assessments of rare variant genotypes beyond categorical autism diagnosis to allow fine-grained phenotypic characterisation.

Earlier research compared autism profiles in individuals with deletions or duplications of 16p11.2 and 22q11.2 to those with idiopathic autism, reporting subtle differences.^24^^,^^30^ Our findings, including a considerably broader range of ND-CNVs, also indicate differences in autism subdomain scores of individuals with ND-CNVs who scored positive for autism (ND-CNVs_ia) compared to those with idiopathic autism, although these were generally not significant. We did, however, find that individuals with 22q11.2 deletion exhibited less impairment in the communication and repetitive behaviour subdomains, which is in line with previous findings.^24^^,^^26^^,^^30^^,^^60^ Regarding comparisons between individuals with SGVs_ia and those with idiopathic autism, we did, uncover differences which indicated relative weaknesses in communication and relative strengths in the repetitive behaviour subdomain in those in the SGVs_ia. Our findings also show that the social subdomain scores of individuals with the SGV *SC2NA* indicated greater impairment compared to those with idiopathic autism. These findings may be partially explained by the large number of non-verbal individuals in the SGVs_ia group.

We found the within-genotype variation in autism domain scores to be greater (73.7–84.6%) than the between-genotype variation (21.4% for the total score, 17.9% for the social, 19.4% for the communication, and 11.8% for the repetitive behaviour subdomain scores). This aligns with and extends previous research comparing autism domains across a more limited number of rare variant genotypes (deletion and duplication at 22q11.2 and 16p11.2).^24^ This goes against the notion that rare variants lead to distinct autism subtypes and generally provides greater evidence of phenotypic convergence rather than divergence across genotypes. The picture is, however, complex, and it should be noted that the between-genotype variation was significant and that the reported effect sizes can be considered large (e.g., eta-squared > 0.14) as defined by Cohen,^61^ indicating that genotype-specific patterns also exist.

The findings have implications for the clinical setting as well as for future research in this area. Clinically, a generalised approach to assessing autism and providing support to individuals with different rare variant genotypes may be useful. Awareness amongst clinicians that, as a group, individuals with a wide range of rare variants have an increased risk of autism will be helpful, as clinical awareness of each individual variant and the associated phenotypic manifestations is often limited.^62^ Yet, our findings of variation in prevalence and subdomain scores across genotypes also indicate the need for genotype-specific support. We detected a sizeable within-genotype variability that has important implications for future research as well as, more down the line, clinically. It suggests that other factors that are not yet well understood are likely to be important in understanding the expressivity of autism phenotypes across rare variant genotypes. Several studies have indicated that common variants with small effect sizes (Odds ratios < 1.1)^2^^,^^63^ can contribute to up to 50% of autism genetic heterogeneity.^64^^,^^65^ This needs to be investigated further in individuals with rare variant genotypes. Additionally, further studies are necessary to assess the likelihood of autism in individuals with rare variants, especially concerning the influence of environmental risk factors like complications during pregnancy^66^ or adverse childhood experiences.^67^ Advances in our understanding of these additional factors and how they impact phenotypic expression in similar or different ways across rare variant genotypes can ultimately lead to more tailored support and care.

In summary, this study detected considerable variation in autism prevalence and subdomain scores across individuals with rare variant genotypes. The greater within-genotype than between-genotype variation indicates a lack of evidence of a solid, distinctive genotype-phenotype correlation between the rare variant genotypes studied and autism. The subdomain scores of individuals with ND-CNVs who scored positive for autism were comparable to those with idiopathic autism. At the same time, those with SGVs who scored positive for autism showed relative weaknesses in communication and relative strengths in the repetitive behaviour subdomain. We recommend that future research assesses individuals with rare genetic variant genotypes, combining standardised clinical autism assessment with detailed and wide-ranging phenotyping (including other neurodevelopmental and mental health conditions, as well as exposure to environmental risk factors) to further increase understanding of autism liability across these genotypes. In addition, the role of common genetic variation (as captured by polygenic risk scores) in the variable expressivity of these rare variants should also be clarified further.^68^ Finally, future research must also focus on assessing the role of other genetic factors, such as disruptive variants elsewhere in the genome, in the prevalence of autism and profile presentation.^9^^,^^17^^,^^69^

### Limitations

This study evaluated autism-related symptomatology using the SCQ, which shows high specificity for autism at the indicated cut-off point of ≥22 and is highly suitable for phenotyping autism in large cohorts.^26^^,^^43^^,^^70^ Nevertheless, it should be noted that a formal diagnosis of autism would require more in-depth clinical diagnostic assessment, such as the ADI-R,^31^ and the findings cannot be generalised to individuals with other rare variant genotypes. It is also important to note that the SCQ has been reported to have reduced accuracy and discriminating capacity in screening for autism in individuals with more profound ID.^71^ The rare variant genotypes examined in this study have all been associated with intellectual disability, although with varying rates and severity. We conducted a sensitivity analysis taking into account the impact of IQ and found our findings remained largely unchanged. However, information on IQ was available in only 701 (53.3%) individuals with rare variant genotypes. Some studies have suggested a higher SCQ cut-off for individuals with more profound ID, and this was one of the reasons we applied the cut-off of ≥22 recommended by the developers of the SCQ, rather than the one of ≥15, which studies have used to screen for autism spectrum disorder.^43^^,^^44^^,^^70^

Another factor to note is that we incorporated items from the SCQ current version for a subset of our cohort for which lifetime data were not available. Previous research has shown that the current version tends to be less accurate than the lifetime version, especially when assessing children with ID.^34^^,^^44^^,^^45^ We recognise that merging data from both versions may introduce confounding bias. Ideally, using the lifetime version throughout would have been preferable, but inclusion of participants from the two sites (The 3q29 project and UCLA cohort) that utilised the current version of the SCQ enabled us to increase the number of young people with rare variants (14.7% contribution) as well as controls (18.6% contribution). Without these sites, we would not have been able to include the very rare 3q29 deletion and duplication genotypes in our analysis, as the 3q29 project contributed 69 out of 74 of these participants (93.2%). Furthermore, inclusion of the current version of the SCQ also enabled this work to benefit from the contribution by UCLA of an enhanced number of young people with the 22q11.2 duplication (n = 79 out of 212 participants (37.3%)). Importantly, it was encouraging that our sensitivity analyses indicated that removing individuals assessed with the current version did not change our findings.

Although, as far as we are aware, we had available the largest possible sample to date to conduct our analyses, it is important to note that for some rare variant genotypes, particularly the SGVs, we were still limited by sample size. This will have impacted our comparisons of individual rare variant genotypes in particular. With increased sample sizes, future studies may find greater evidence of differences in autism prevalence and subdomain scores between genotypes.

It is also important to consider the effect of ascertainment bias. Although we took this into account to a degree by covarying for study site, differences in referral for genetic testing between those with ND-CNVs and SGVs may still have impacted our comparisons of these individuals. It should be emphasised, though, that the considerable differences in prevalence and subdomain scores we found between the different rare variant genotypes (including within ND-CNVs and SGVs) are unlikely to be explained by ascertainment bias alone. Furthermore, all participants in this study had a genetic diagnosis. As not all individuals with rare variant genotypes will have phenotypic manifestations deemed sufficiently severe to warrant referral for genetic testing, this work may overestimate the true impact of autism on those with rare variant genotypes.^8^^,^^24^^,^^26^

### Conclusion

The presence of a rare genetic variant genotype was associated with a 43 times increased risk of autism, and autism prevalence and subdomain scores were found to vary considerably across genotypes. However, the variation in autism total and subdomain scores within genotypes was considerably greater than between genotypes, indicating lack of strong evidence that rare variant genotypes are linked with discrete autism behavioural phenotypes. This implies that the specific rare variant—on its own—has limited autism phenotype predictability and prognostic significance, warranting the need for better understanding of the role other factors (both genetic and environmental) to move towards tailored clinical management of young people with rare variants.

## Contributors

MBMvdB acquired the funding for the project and supervised the project. NMHA and MBMvdB arranged and coordinated data collection. NMHA, SJRAC, MBMvdB, and MJO were involved in the analysis and interpretation of the data. NMHA, SJRAC, MBMvdB, and MJO drafted the manuscript, which the other authors revised. LKW and CEB contributed to data collection from UCLA and reviewed the manuscript. JGM and RMP contributed to data collection from Rutgers University and reviewed the manuscript. REG reviewed the manuscript. WKC contributed to data collection from Simon's Searchlight and reviewed the manuscript. The IMAGINE-ID consortium contributed to the data collection from Cardiff University. NMHA, SJRAC, and MBMvdB directly accessed and verified the underlying data. All authors read and approved the final version of the manuscript.

## Data sharing statement

The data used in this study is available upon request. Please contact Prof. van den Bree (vandenbreemb@cardiff.ac.uk) for any data requests. Approved researchers can obtain the Simons Searchlight population dataset described in this study (https://www.sfari.org/resource/simons-searchlight/) by applying at https://base.sfari.org. Approved researchers can obtain the SPARK population dataset described in this study https://www.sfari.org/resource/spark/ by applying at https://base.sfari.org. The full phenotypic IMAGINE dataset is available from the UK Data Archive under special licence access (SN 8621). Requests for genotype or linked genotypic-phenotypic data can be made through the study's data access committee: https://imagine-id.org/healthcare-professionals/datasharing/.

## Declaration of interests

MJO and MBMvdB report grants from Takeda Pharmaceuticals outside of the submitted work. MJO reports a grant from Akrivia Health outside of the submitted work. CEB reports participation in a One Mind Scientific Advisory Board that is unrelated to the content of this manuscript. All other authors declare no competing interests.

## Appendix A Supplementary data

Supplementary Material

IMAGINE ID Consortioum Members

## References

[1] American Psychiatric Association (2013).

[2] Anney R., Klei L., Pinto D. (2012). Individual common variants exert weak effects on the risk for autism spectrum disorders. Hum Mol Genet.

[3] Klei L., McClain L.L., Mahjani B. (2021). How rare and common risk variation jointly affect liability for autism spectrum disorder. Mol Autism.

[4] Sebat J., Lakshmi B., Malhotra D. (2007). Strong association of de novo copy number mutations with autism. Science.

[5] Havdahl A., Niarchou M., Starnawska A., Uddin M., van der Merwe C., Warrier V. (2021). Genetic contributions to autism spectrum disorder. Psychol Med.

[6] Marshall C.R., Noor A., Vincent J.B. (2008). Structural variation of chromosomes in autism spectrum disorder. Am J Hum Genet.

[7] Vicari S., Napoli E., Cordeddu V. (2019). Copy number variants in autism spectrum disorders. Prog Neuropsychopharmacol Biol Psychiatry.

[8] Raznahan A., Won H., Glahn D.C., Jacquemont S. (2022). Convergence and divergence of rare genetic disorders on brain phenotypes: a review. JAMA Psychiatry.

[9] Gilman S.R., Iossifov I., Levy D., Ronemus M., Wigler M., Vitkup D. (2011). Rare de novo variants associated with autism implicate a large functional network of genes involved in formation and function of synapses. Neuron.

[10] Huguet G., Benabou M., Bourgeron T., Sassone-Corsi P., Christen Y. (2016). A Time for Metabolism and Hormones.

[11] Kirov G. (2015). CNVs in neuropsychiatric disorders. Hum Mol Genet.

[12] Takumi T., Tamada K. (2018). CNV biology in neurodevelopmental disorders. Curr Opin Neurobiol.

[13] Rees E., Kirov G. (2021). Copy number variation and neuropsychiatric illness. Curr Opin Genet Dev.

[14] Huguet G., Ey E., Bourgeron T. (2013). The genetic landscapes of autism spectrum disorders. Annu Rev Genom Hum Genet.

[15] Iossifov I., Ronemus M., Levy D. (2012). De novo gene disruptions in children on the autistic spectrum. Neuron.

[16] Mollon J., Almasy L., Jacquemont S., Glahn D.C. (2023). The contribution of copy number variants to psychiatric symptoms and cognitive ability. Mol Psychiatry.

[17] Rylaarsdam L., Guemez-Gamboa A. (2019). Genetic causes and modifiers of autism spectrum disorder. Front Cell Neurosci.

[18] Molloy C.J., Quigley C., McNicholas Á., Lisanti L., Gallagher L. (2023). A review of the cognitive impact of neurodevelopmental and neuropsychiatric associated copy number variants. Transl Psychiatry.

[19] Jacquemont S., Huguet G., Klein M. (2022). Genes to mental health (G2MH): a framework to map the combined effects of rare and common variants on dimensions of cognition and psychopathology. Am J Psychiatry.

[20] Cunningham A.C., Hall J., Owen M.J., van den Bree M.B.M. (2021). Coordination difficulties, IQ and psychopathology in children with high-risk copy number variants. Psychol Med.

[21] Toma C. (2020). Genetic variation across phenotypic severity of autism. Trends Genet.

[22] Binder E.B. (2021). Genotype-phenotype predictions in autism: are we there yet?. Am J Psychiatry.

[23] Richards C., Jones C., Groves L., Moss J., Oliver C. (2015). Prevalence of autism spectrum disorder phenomenology in genetic disorders: a systematic review and meta-analysis. Lancet Psychiatry.

[24] Chawner S., Doherty J.L., Anney R.J.L. (2021). A genetics-first approach to dissecting the heterogeneity of autism: phenotypic comparison of autism risk copy number variants. Am J Psychiatry.

[25] Bozhilova N., Welham A., Adams D. (2023). Profiles of autism characteristics in thirteen genetic syndromes: a machine learning approach. Mol Autism.

[26] Chawner S.J.R.A., Owen M.J., Holmans P. (2019). Genotype–phenotype associations in children with copy number variants associated with high neuropsychiatric risk in the UK (IMAGINE-ID): a case-control cohort study. Lancet Psychiatry.

[27] Bruining H., Eijkemans M.J., Kas M.J., Curran S.R., Vorstman J.A., Bolton P.F. (2014). Behavioral signatures related to genetic disorders in autism. Mol Autism.

[28] Bruining H., de Sonneville L., Swaab H. (2010). Dissecting the clinical heterogeneity of autism spectrum disorders through defined genotypes. PLoS One.

[29] Lee N.R., Niu X., Zhang F. (2022). Variegation of autism related traits across seven neurogenetic disorders. Transl Psychiatry.

[30] Serur Y., Sofrin Frumer D., Daon K. (2019). Psychiatric disorders and autism in young children with 22q11.2 deletion syndrome compared to children with idiopathic autism. Eur Psychiatry.

[31] Lord C., Rutter M., Le Couteur A. (1994). Autism Diagnostic Interview-Revised: a revised version of a diagnostic interview for caregivers of individuals with possible pervasive developmental disorders. J Autism Dev Disord.

[32] Eaves L.C., Wingert H.D., Ho H.H., Mickelson E.C.R. (2006). Screening for autism spectrum disorders with the social communication questionnaire. J Dev Behav Pediatr.

[33] Charman T., Baird G., Simonoff E. (2007). Efficacy of three screening instruments in the identification of autistic-spectrum disorders. Br J Psychiatry.

[34] Chesnut S.R., Wei T., Barnard-Brak L., Richman D.M. (2017). A meta-analysis of the social communication questionnaire: screening for autism spectrum disorder. Autism.

[35] Chandler S., Charman T., Baird G. (2007). Validation of the social communication questionnaire in a population cohort of children with autism spectrum disorders. J Am Acad Child Adolesc Psychiatry.

[36] Pollak R.M., Murphy M.M., Epstein M.P. (2019). Neuropsychiatric phenotypes and a distinct constellation of ASD features in 3q29 deletion syndrome: results from the 3q29 registry. Mol Autism.

[37] Pollak R.M., Zinsmeister M.C., Murphy M.M., Zwick M.E., Mulle J.G. (2020). New phenotypes associated with 3q29 duplication syndrome: results from the 3q29 registry. Am J Med Genet A.

[38] The Simons Vip Consortium (2012). Simons Variation in Individuals Project (Simons VIP): a genetics-first approach to studying autism spectrum and related neurodevelopmental disorders. Neuron.

[39] Arpi M.N.T., Simpson T.I. (2022). SFARI genes and where to find them; modelling Autism Spectrum Disorder specific gene expression dysregulation with RNA-seq data. Sci Rep.

[40] Abrahams B.S., Arking D.E., Campbell D.B. (2013). SFARI Gene 2.0: a community-driven knowledgebase for the autism spectrum disorders (ASDs). Mol Autism.

[41] Feliciano P., Daniels A.M., Green Snyder L. (2018). SPARK: a US cohort of 50,000 families to accelerate autism research. Neuron.

[42] Lin A., Vajdi A., Kushan-Wells L. (2020). Reciprocal copy number variations at 22q11.2 produce distinct and convergent neurobehavioral impairments relevant for schizophrenia and autism spectrum disorder. Biol Psychiatry.

[43] Allen C.W., Silove N., Williams K., Hutchins P. (2007). Validity of the social communication questionnaire in assessing risk of autism in preschool children with developmental problems. J Autism Dev Disord.

[44] Hollocks M.J., Casson R., White C., Dobson J., Beazley P., Humphrey A. (2019). Brief report: an evaluation of the social communication questionnaire as a screening tool for autism spectrum disorder in young people referred to child & adolescent mental health services. J Autism Dev Disord.

[45] Wei T., Chesnut S.R., Barnard-Brak L., Richman D. (2015). Psychometric analysis of the social communication questionnaire using an item-response theory framework: implications for the use of the lifetime and current forms. J Psychopathol Behav Assess.

[46] Axelrod B.N. (2002). Validity of the Wechsler abbreviated scale of intelligence and other very short forms of estimating intellectual functioning. Assessment.

[47] Puttaswamy A., Barone A., Viezel K.D., Willis J.O., Dumont R. (2020). Ancillary and Complementary Index Critical Values and Base Rates for the Normative Sample. J Psychoeduc Assess.

[48] D'Angelo D., Lebon S., Chen Q. (2016). Defining the effect of the 16p11.2 duplication on cognition, behavior, and medical comorbidities. JAMA Psychiatry.

[49] Meteyard L., Davies R.A.I. (2020). Best practice guidance for linear mixed-effects models in psychological science. J Mem Lang.

[50] Niarchou M., Chawner S., Doherty J.L. (2019). Psychiatric disorders in children with 16p11.2 deletion and duplication. Transl Psychiatry.

[51] Midway S., Robertson M., Flinn S., Kaller M. (2020). Comparing multiple comparisons: practical guidance for choosing the best multiple comparisons test. PeerJ.

[52] Richardson J.T.E. (2011). Eta squared and partial eta squared as measures of effect size in educational research. Educ Res Rev.

[53] Cunningham A.C., Delport S., Cumines W. (2018). Developmental coordination disorder, psychopathology and IQ in 22q11.2 deletion syndrome. Br J Psychiatry.

[54] Seitz-Holland J., Lyons M., Kushan L. (2021). Opposing white matter microstructure abnormalities in 22q11.2 deletion and duplication carriers. Transl Psychiatry.

[55] Wenger T.L., Miller J.S., DePolo L.M. (2016). 22q11.2 duplication syndrome: elevated rate of autism spectrum disorder and need for medical screening. Mol Autism.

[56] Malhotra D., Sebat J. (2012). CNVs: harbingers of a rare variant revolution in psychiatric genetics. Cell.

[57] Rosenfeld J.A., Coe B.P., Eichler E.E., Cuckle H., Shaffer L.G. (2013). Estimates of penetrance for recurrent pathogenic copy-number variations. Genet Med.

[58] Arnett A.B., Rhoads C.L., Hoekzema K. (2018). The autism spectrum phenotype in ADNP syndrome. Autism Res.

[59] Dang T., Duan W.Y., Yu B. (2018). Autism-associated Dyrk1a truncation mutants impair neuronal dendritic and spine growth and interfere with postnatal cortical development. Mol Psychiatry.

[60] Selten I., Boerma T., Everaert E. (2023). Behaviors related to autism spectrum disorder in children with developmental language disorder and children with 22q11.2 deletion syndrome. Autism Dev Lang Impair.

[61] Cohen J. (1988).

[62] Chawner S.J., Watson C.J., Owen M.J. (2021). Clinical evaluation of patients with a neuropsychiatric risk copy number variant. Curr Opin Genet Dev.

[63] Bray N.J., O'Donovan M.C. (2019). The genetics of neuropsychiatric disorders. Brain Neurosci Adv.

[64] Chaste P., Roeder K., Devlin B. (2017). The Yin and Yang of autism genetics: how rare de novo and common variations affect liability. Annu Rev Genom Hum Genet.

[65] Gaugler T., Klei L., Sanders S.J. (2014). Most genetic risk for autism resides with common variation. Nat Genet.

[66] Lu J., Wang Z., Liang Y., Yao P. (2022). Rethinking autism: the impact of maternal risk factors on autism development. Am J Transl Res.

[67] Baldwin J.R., Sallis H.M., Schoeler T. (2023). A genetically informed registered report on adverse childhood experiences and mental health. Nat Human Behav.

[68] Niemi M.E.K., Martin H.C., Rice D.L. (2018). Common genetic variants contribute to risk of rare severe neurodevelopmental disorders. Nature.

[69] Wiśniowiecka-Kowalnik B., Nowakowska B.A. (2019). Genetics and epigenetics of autism spectrum disorder—current evidence in the field. J Appl Genet.

[70] Charman T., Gotham K. (2013). Measurement issues: screening and diagnostic instruments for autism spectrum disorders – lessons from research and practise. Child Adolesc Ment Health.

[71] Sappok T., Diefenbacher A., Gaul I., Bölte S. (2015). Validity of the social communication questionnaire in adults with intellectual disabilities and suspected autism spectrum disorder. Am J Intellect Dev Disabil.

