# Supplementary material for: Comparison of autism domains across thirty rare variant genotypes
Source: eBioMedicine. 2025 Jan 31;112:105521. doi: 10.1016/j.ebiom.2024.105521 (PMC11835590; doi:10.1016/j.ebiom.2024.105521)
Supplement: Supplementary Material [file mmc1.docx]

Table of Contents

[Supplementary Table 1 Detailed information on the ND-CNV cohort 2](#_Toc183604338)

[Supplementary Table 2 Information on other conditions associated with autism and the use of medication for individuals with rare variant genotypes and controls*. 3](#_Toc183604339)

[Supplementary Table 3 Research findings at a SCQ cut-off >=15 compared to a cut-off >=22 4](#_Toc183604340)

[Supplementary Table 4 Comparison of indicative autism prevalence between individuals with rare variant genotypes and controls 5](#_Toc183604341)

[Supplementary Table 5 Comparison of indicative autism prevalence across individuals with rare variant genotypes 6](#_Toc183604342)

[Supplementary Table 6 Autism total and subdomain scores in individuals with ND-CNVs compared to those with SGVs 7](#_Toc183604343)

[Supplementary Table 7 Autism total and subdomain scores for the 30 rare variant genotypes 8](#_Toc183604344)

[Supplementary Table 8 Variation in autism total and subdomain scores explained between and within rare variant genotypes 9](#_Toc183604345)

[Supplementary Table 9 Autism total and subdomain scores for individuals with rare variant genotypes who scored positive on the SCQ compared to individuals with idiopathic autism 10](#_Toc183604346)

[Supplementary Table 10 Comparison of autism subdomain scores in individuals with rare variant genotypes who scored above the cutoff point (>=22) for indicative autism versus those with idiopathic autism 11](#_Toc183604347)

[Supplementary Table 11 Indicative autism prevalence total and subdomain scores in rare variant genotypes after accounting for background, income, other conditions*, and medication use. 12](#_Toc183604348)

[Supplementary Table 12 Comparison of indicative autism prevalence in individuals with rare variant genotypes and controls after accounting for FSIQ, VIQ and PIQ 13](#_Toc183604349)

[Supplementary Table 13 Comparison of indicative autism prevalence, total and subdomain scores in individuals with rare variant genotypes and controls after excluding the SCQ current version. 14](#_Toc183604350)

[Supplementary Table 14 Comparison of indicative autism prevalence, total and subdomain scores in individuals with rare variant genotypes after excluding nonverbal individuals. 15](#_Toc183604351)

# Supplementary Table 1 Detailed information on the ND-CNV cohort

| ND-CNV cohort | Sample size | Sex  Male  N (%) | Age  mean±SD | Indicative autism  N (%) * |
| --- | --- | --- | --- | --- |
| ND-CNV cohort (N=1005) | | | | |
| Cardiff University cohort (N=493) | | | | |
| 1q21.1 deletion | 19 | 13 (68) | 9.1 ± 2.6 | 5 (26) |
| 1q21.1 duplication | 31 | 17 (55) | 9.3 ± 3.1 | 16 (52) |
| 1q21.1 TAR duplication | 14 | 8 (57) | 8.5± 2.7 | 4 (29) |
| 2p16.3 deletion *NRXN1* deletion | 19 | 16(84) | 8.4 ± 2.7 | 8 (42) |
| 3q29 deletion | 5 | 4 (80) | 9.2± 2.8 | 2 (40) |
| 9q34.4 deletion Kleefstra syndrome | 13 | 5 (38) | 11.6± 3.9 | 7 (54) |
| 15q13.3 deletion | 27 | 21 (78) | 9.7 ± 3.8 | 11 (41) |
| 15q13.3 duplication | 23 | 15 (65) | 9.4± 3.2 | 8 (35) |
| 15q11.2 deletion | 42 | 32 (76) | 9. 2± 3.1 | 16 (38) |
| 16p11.2 deletion | 59 | 38 (64) | 9.5 ±3.3 | 17 (29) |
| 16p11.2 distal deletion | 12 | 8 (67) | 9.8 ±3.6 | 5 (42) |
| 16p11.2 duplication | 32 | 20(62) | 10.2 ± 3.6 | 12 (38) |
| 22q11.2 deletion | 164 | 90 (55) | 9.5± 2.6 | 22 (13) |
| 22q11.2 duplication | 33 | 23 (70) | 9.7± 3.6 | 11 (33) |
| Simons Searchlight cohort (N=364) | | | | |
| 1q21.1 deletion | 28 | 17 (61) | 7.6± 4.0 | 9 (32) |
| 1q21.1 duplication | 36 | 22 (61) | 8.2 ± 3.5 | 16 (44) |
| 16p11.2 deletion | 195 | 108 (55) | 8.6± 3.7 | 31 (16) |
| 16p11.2 distal deletion | 12 | 6 (50) | 8.9± 2.7 | 4 (33) |
| 16p11.2 duplication | 93 | 56 (60) | 8.2± 3.6 | 27 (29) |
| The 3q29 Project cohort (N=69) | | | | |
| 3q29 deletion | 56 | 35 (62) | 9.2± 3.8 | 12 (21) |
| 3q29 duplication | 13 | 5 (38) | 8.5± 3.3 | 6 (46) |
| UCLA cohort (N=79) | | | | |
| 22q11.2 deletion | 48 | 24 (50) | 12.3± 3.6 | 0 (0) |
| 22q11.2 duplication | 31 | 16 (52) | 10.6± 3.1 | 3 (10) |

* Based on a SCQ >=22

# Supplementary Table 2 Information on other conditions associated with autism and the use of medication for individuals with rare variant genotypes and controls*.

|  | Controls | | ND-CNVs | | SGVs | |
| --- | --- | --- | --- | --- | --- | --- |
|  | **N with complete data** | **N (%)** | **N with complete data** | **N (%)** | **N with complete data** | **N (%)** |
| Conditions | | | | | | |
| ADHD | 318 | 34 (10.7) | 794 | 339 (42.7) | 32 | 31 (96.9) |
| Any anxiety | 358 | 32 (8.9) | 838 | 272 (32.5) | 14 | 13 (92.8) |
| Seizures | 205 | 1 (0.5) | 510 | 95 (18.6) | 208 | 134 (64.4) |
| Sleep problems | 366 | 66 (18) | 786 | 247 (31.4) | 180 | 100 (55) |
| Oppositional defiant disorder | 246 | 11 (4.5) | 604 | 112 (18.5) | 3 | 2 (66.7) |
| Conduct disorder | 277 | 2 (0.7) | 630 | 8 (1.3) | 1 | 0(0) |
| Depression | 291 | 5 (1.7) | 685 | 21 (3.1) | 1 | 0(0) |
| Tic disorder | 354 | 3 (0.8) | 960 | 95 (9.9) | 256 | 19 (7.5) |
| Medications | | | | | | |
| Anticonvulsants | 50 | 1(2) | 233 | 74 (31.7) | 136 | 128 (94.1) |
| Antidepressants | 95 | 1(1.0) | 608 | 36(5.9) | 25 | 25(100) |
| Anxiolytics | 95 | 1(1.0) | 590 | 22(5.9) | 36 | 35(97.2) |
| Sleep medications | 212 | 3(1.4) | 642 | 112(17.4) | 12 | 11(91.2) |
| Stimulants | 100 | 4(4) | 623 | 96(15.4) | 27 | 27(100) |
| Antipsychotics | 46 | 0(0) | 177 | 22(12.4) | 18 | 12(66.7) |

*This information was not available to all participants

# Supplementary Table 3 Research findings at a SCQ cut-off >=15 compared to a cut-off >=22

|  | Cut-off >=22 | Cut-off >=15 |
| --- | --- | --- |
| Indicative autism in young people with a rare variant versus controls | 32% vs 2% (OR= 43.1, CI= 6.6–282.2, p<0.001) | 45.3% vs 2% (OR= 53.1, CI= 7.7–367.4, p<0.001) |
| Indicative autism in young people with SGVs versus ND-CNVs | 53% vs 25% (OR=4.00, CI=2.2–7.3, p=0.002) | 75.7% vs 48.3% (OR=4.8, CI= 2.1-6.4, p <0.0001) |
| Variation across the 30 rare variant groups | Range 10-85% | Range 28.3 – 100% |

# Supplementary Table 4 Comparison of indicative autism prevalence between individuals with rare variant genotypes and controls

| Rare Variant Genotypes | Odds Ratio | CI | P  (mixed effects logistic regression)* |
| --- | --- | --- | --- |
| Rare variant groups | | | |
| ND-CNVs & SGVs | 43.1 | 6.6-282.2 | **<0.0001** |
| ND-CNVs | 22.73 | 5.88 – 87.89 | **<0.0001** |
| SGVs | 99.59 | 24.45 – 405.69 | **<0.0001** |
| Individual rare variant genotypes | | | |
| 22q11.2 deletion | 4.19 | 1.94 – 9.02 | **0.042** |
| SETBP1 | 16.18 | 2.62 – 100.02 | 0.442 |
| 16p11.2 deletion | 12.94 | 6.29 – 26.64 | <**0.0001** |
| HNRNPH2 | 122.35 | 25.94 – 577.01 | **0.002** |
| HIVEP2 | 25.82 | 5.13 – 129.95 | **0.018** |
| 3q29 deletion | 17.82 | 7.03 – 45.18 | **<0.0001** |
| 1q21.1 TAR duplication | 15.03 | 3.50 – 64.64 | 0.063 |
| 22q11.2 duplication | 12.16 | 4.94 – 29.92 | **<0.0001** |
| SLC6A1 | 25.37 | 7.86 – 81.88 | **<0.0001** |
| CTNNB1 | 48.85 | 12.73 – 187.43 | **<0.0001** |
| 1q21.1 deletion | 22.98 | 8.84 – 59.75 | **<0.0001** |
| PACS1 | 66.71 | 17.91 – 248.51 | **<0.0001** |
| 16p11.2 duplication | 27.07 | 12.28 – 59.70 | **<0.0001** |
| PPP2R5D | 71.00 | 24.16 – 208.63 | **<0.0001** |
| 16p11.2 distal deletion | 30.66 | 9.85 – 95.40 | **<0.0001** |
| STXBP1 | 294.91 | 82.78 – 1050.65 | **<0.0001** |
| 15q13.3 duplication | 18.99 | 5.93 – 60.84 | **<0.0001** |
| SYNGAP1 | 87.40 | 26.15 – 292.14 | **<0.0001** |
| 15q11.2 deletion | 20.50 | 7.86 – 53.43 | **<0.0001** |
| 15q13.3 deletion | 23.75 | 7.93 – 71.06 | **<0.0001** |
| NRXN1 deletion | 25.04 | 7.30 – 85.94 | **0.0001** |
| GRIN2B | 98.54 | 31.33 – 309.89 | **<0.0001** |
| 1q21.1 duplication | 52.42 | 22.01 – 124.86 | **<0.0001** |
| MED13L | 80.36 | 17.25 – 374.41 | **<0.0001** |
| 3q29 duplication | 78.50 | 18.64 – 330.67 | **<0.0001** |
| Kleefstra syndrome | 47.89 | 11.83 – 193.79 | **<0.0001** |
| SCN2A | 141.85 | 51.82 – 388.31 | **<0.0001** |
| ASXL3 | 218.65 | 57.28 – 834.56 | **<0.0001** |
| DYRK1A | 493.24 | 80.70 – 3014.48 | **<0.0001** |
| ADNP | 489.78 | 80.08 – 2995.41 | **<0.0001** |

* P-values were corrected for multiple comparisons using Tukey adjustment. Findings remained significant following sensitivity analyses, taking into account ethnicity, income, other conditions associated with autism, and medication use.

# Supplementary Table 5 Comparison of indicative autism prevalence across individuals with rare variant genotypes

| **Rare variant genotypes** | **Estimate** | **Standard error** | **Z ratio** | **p (mixed effects logistic regression)** |
| --- | --- | --- | --- | --- |
| 22q11.2_Del - *ADNP* | -4.76 | 0.905 | -5.25 | **<0.0001** |
| 22q11.2_Del - *DYRK1A* | -4.76 | 0.905 | -5.26 | **<0.0001** |
| 22q11.2_Del - *STXBP1* | -4.25 | 0.622 | -6.83 | **<0.0001** |
| 22q11.2_Del - *ASXL3* | -3.95 | 0.659 | -5.99 | **<0.0001** |
| 22q11.2_Del - *SCN2A* | -3.52 | 0.482 | -7.31 | **<0.0001** |
| 22q11.2_Del - *GRIN2B* | -3.16 | 0.558 | -5.66 | **<0.0001** |
| 22q11.2_Del - *HNRNPH2* | -3.37 | 0.772 | -4.37 | **0.004** |
| 22q11.2_Del - *PPP2R5D* | -2.83 | 0.521 | -5.42 | **<0.0001** |
| 22q11.2_Del - *SYNGAP1* | -3.04 | 0.590 | -5.14 | **<0.0001** |
| 22q11.2_Del - *PACS1* | -2.77 | 0.648 | -4.27 | **0.0073** |
| 22q11.2_Del - *MED13L* | -2.95 | 0.765 | -3.86 | **0.0362** |
| 22q11.2_Del - 3q29_Dup | -2.93 | 0.713 | -4.11 | **0.0140** |
| 1q21.1_Del - 22q11.2_Del | 1.70 | 0.448 | 3.79 | **0.0444** |
| 1q21.1_Del - *STXBP1* | -2.55 | 0.633 | -4.02 | **0.0191** |
| 1q21.1_Dup - 22q11.2_Del | 2.52 | 0.397 | 6.36 | **<0.0001** |
| 16p11.2_Del - *SCN2A* | -2.39 | 0.397 | -6.02 | **<0.0001** |
| 16p11.2_Del - *STXBP1* | -3.13 | 0.555 | -5.63 | **<0.0001** |
| 16p11.2_Del - *DYRK1A* | -3.64 | 0.859 | -4.23 | **0.0085** |
| 16p11.2_Del - *ADNP* | -3.63 | 0.860 | -4.22 | **0.0089** |
| 16p11.2_Del - *GRIN2B* | -2.02 | 0.490 | -4.14 | **0.0123** |
| 16p11.2_Del - 1q21.1_Dup | -1.39 | 0.341 | -4.097 | **0.0147** |
| 16p11.2_Del - *ASXL3* | -2.82 | 0.598 | -4.72 | **<0.0001** |
| 16p11.2_Dup - 22q11.2_Del | 1.87 | 0.360 | 5.18 | **<0.0001** |
| 16p11.2_Dup - *STXBP1* | -2.39 | 0.556 | -4.28 | **0.0067** |
| 16p11.2_Dup - *SCN2A* | -1.66 | 0.405 | -4.08 | **0.0154** |
| 22q11.2_Dup - *ADNP* | -3.69 | 0.916 | -4.03 | **0.018** |
| 22q11.2_Dup - *ASXL3* | -2.89 | 0.675 | -4.27 | **0.0071** |
| 22q11.2_Dup - *DYRK1A* | -3.70 | 0.915 | -4.04 | **0.0181** |
| 22q11.2_Dup - *STXBP1* | -3.19 | 0.638 | -4.99 | **<0.0001** |
| 22q11.2_Dup - *SCN2A* | -2.46 | 0.507 | -4.844 | **<0.0001** |
| 3q29_Del - *ASXL3* | -2.51 | 0.656 | -3.81 | **0.0416** |
| 3q29_Del - *STXBP1* | -2.80 | 0.617 | -4.54 | **0.002** |
| 3q29_Del - *SCN2A* | -2.07 | 0.484 | -4.30 | **0.006** |

The table shows post-hoc comparisons of autism trait prevalence across rare variant genotypes. P-values were corrected for multiple comparisons using Tukey adjustment, and only comparisons showing significant differences were included in the table.

# Supplementary Table 6 Autism total and subdomain scores in individuals with ND-CNVs compared to those with SGVs

| Autism total and subdomain scores | **ND-CNVs (N=1005)**  **Mean ± SD** | **SGVs (N=309)**  **Mean ± SD** | **Group contrast estimates (CI)** | **p (mixed effects linear regression) *** |
| --- | --- | --- | --- | --- |
| Total score | 14.9 ± 8.7 | 21.1 ± 8.3 | 6.9 (4.6-9.3) | **<0.001** |
| Social subdomain score | 7.3 ± 5.2 | 10.4 ± 5.4 | 3.9 (2.6-5.3) | **<0.001** |
| Communication subdomain score | 4.4 ± 2.6 | 6.3 ± 2.4 | 1.9 (1.31-2.6) | **<0.001** |
| Repetitive behaviour subdomain score | 3.2 ± 2.5 | 4.2 ± 2.2 | 0.9 (0.3-1.4) | **0.009** |

* P-values were corrected for multiple comparisons using Tukey adjustment. Findings remained significant following sensitivity analyses accounting for ethnicity, income, other conditions associated with autism, and medication use.

N refers to the number of participants; SD refers to the standard deviation

# Supplementary Table 7 Autism total and subdomain scores for the 30 rare variant genotypes

|  |  | Social  subdomain score | | Communication  subdomain score | | Repetitive behaviour  subdomain score | | Total  score | |
| --- | --- | --- | --- | --- | --- | --- | --- | --- | --- |
| Rare variant genotypes | N | Mean | SD | Mean | SD | Mean | SD | Mean | SD |
| 1q21.1 deletion | 47 | 7.09 | 5.34 | 4.32 | 2.60 | 3.66 | 2.43 | 15.09 | 8.39 |
| 1q21.1 duplication | 67 | 9.00 | 5.72 | 5.24 | 2.67 | 4.54 | 2.68 | 18.92 | 10.07 |
| 1q21.1 TAR duplication | 14 | 7.57 | 5.23 | 3.19 | 1.62 | 3.36 | 2.79 | 14.28 | 8.00 |
| 2p16.3 deletion (*NRXN1*) | 19 | 10.95 | 4.36 | 5.74 | 1.82 | 4.58 | 2.36 | 21.34 | 7.14 |
| 3q29 deletion | 61 | 6.67 | 4.55 | 4.51 | 2.79 | 3.23 | 2.52 | 14.46 | 8.05 |
| 3q29 duplication | 13 | 9.23 | 5.66 | 5.86 | 3.25 | 4.08 | 2.33 | 19.37 | 10.13 |
| 9q34.4 deletion (Kleefstra) | 13 | 10.77 | 5.51 | 5.97 | 2.15 | 4.69 | 1.65 | 21.15 | 7.63 |
| 15q11.2 deletion | 42 | 10.00 | 5.12 | 5.69 | 2.30 | 4.50 | 2.13 | 20.21 | 7.61 |
| 15q13.3 deletion | 27 | 9.70 | 5.47 | 5.18 | 2.59 | 3.41 | 2.41 | 18.31 | 8.60 |
| 15q13.3 duplication | 23 | 8.57 | 5.27 | 4.79 | 3.14 | 3.78 | 2.58 | 17.16 | 9.27 |
| 16p11.2 deletion | 254 | 6.29 | 5.32 | 4.21 | 2.51 | 2.95 | 2.37 | 13.47 | 8.54 |
| 16p11.2 distal deletion | 24 | 8.75 | 4.53 | 5.77 | 2.25 | 3.62 | 2.45 | 18.10 | 7.58 |
| 16p11.2 duplication | 125 | 7.53 | 5.18 | 4.91 | 2.62 | 3.81 | 2.58 | 16.29 | 9.10 |
| 22q11.2 deletion | 212 | 6.42 | 4.34 | 3.24 | 2.10 | 2.07 | 2.09 | 11.74 | 6.93 |
| 22q11.2 duplication | 64 | 6.70 | 4.83 | 4.14 | 3.00 | 3.12 | 2.67 | 13.96 | 8.94 |
| *ADNP* | 13 | 14.31 | 4.52 | 8.11 | 1.14 | 5.38 | 1.85 | 28.29 | 6.04 |
| *ASXL3* | 20 | 12.06 | 5.21 | 5.47 | 2.90 | 5.30 | 1.63 | 22.45 | 6.92 |
| *CTNNB1* | 16 | 9.56 | 4.43 | 4.12 | 2.31 | 5.00 | 2.53 | 20.03 | 7.60 |
| *DYRK1A* | 13 | 12.85 | 3.67 | 6.83 | 1.80 | 5.15 | 2.03 | 25.32 | 5.62 |
| *GRIN2B* | 27 | 10.78 | 5.34 | 6.53 | 2.31 | 3.67 | 2.20 | 21.04 | 6.92 |
| *HIVEP2* | 11 | 7.45 | 4.72 | 5.95 | 2.19 | 3.73 | 1.95 | 17.12 | 7.34 |
| *HNRNPH2* | 11 | 10.27 | 4.47 | 6.36 | 2.17 | 3.18 | 1.78 | 19.93 | 7.11 |
| *MED13L* | 11 | 9.64 | 5.89 | 6.45 | 2.21 | 5.09 | 2.63 | 21.05 | 9.23 |
| *PACS1* | 17 | 8.71 | 3.95 | 6.25 | 1.94 | 4.53 | 2.21 | 19.52 | 6.33 |
| *PPP2R5D* | 33 | 8.21 | 6.13 | 5.25 | 2.52 | 4.06 | 2.19 | 17.82 | 9.44 |
| *SCN2A* | 52 | 12.58 | 5.29 | 6.65 | 2.44 | 3.37 | 2.43 | 23.34 | 8.31 |
| *SETBP1* | 10 | 6.50 | 4.14 | 6.24 | 2.45 | 4.20 | 2.10 | 16.60 | 7.10 |
| *SLC6A1* | 27 | 6.89 | 4.60 | 4.89 | 2.18 | 3.74 | 2.23 | 15.47 | 7.53 |
| *STXBP1* | 26 | 12.12 | 4.23 | 7.72 | 1.57 | 4.27 | 2.01 | 24.20 | 6.25 |
| *SYNGAP1* | 22 | 9.05 | 5.38 | 5.49 | 2.43 | 4.68 | 2.08 | 19.40 | 8.83 |

# Supplementary Table 8 Variation in autism total and subdomain scores explained between and within rare variant genotypes

|  | N | Sum Squares | Mean  Square | Degrees of freedom | F  statistic | Cohen coefficient | Between-genotype variation | | Within-genotype variation  *** | Variation explained by covariates  **** |
| --- | --- | --- | --- | --- | --- | --- | --- | --- | --- | --- |
|  |  |  |  |  |  |  | ɳ²* | p** |  |  |
| Total score | | | | | | | | | | |
| Rare variant genotype | 1314 | 23.49 | 809.9 | 29 | 12.8 | 0.54 | 21.4 | **<0.0001** | 73.7 | 4.9 |
| Social subdomain | | | | | | | | | | |
| Rare variant genotype | 1314 | 7047 | 243.0 | 29 | 10.6 | 0.49 | 17.9 | **<0.0001** | 75.0 | 7.1 |
| Communication subdomain | | | | | | | | | | |
| Rare variant genotype | 1314 | 1846 | 63.7 | 29 | 10.9 | 0.49 | 19.4 | **<0.0001** | 78.8 | 1.8 |
| Repetitive behaviour subdomain | | | | | | | | | | |
| Rare variant genotype | 1314 | 936 | 32.3 | 29 | 6.1 | 0.37 | 11.7 | **<0.0001** | 84.6 | 3.6 |

* ɳ²: eta-squared, the proportion of variance in the score that is explained by rare variant genotype (between-genotype variation)

****** p-value: the significance associated with the between-group variation

*** Variation remaining within each variant genotype after taking into account the between-genotype variation and variation explained by other covariates

**** Covariates: age, gender, and study site

# Supplementary Table 9 Autism total and subdomain scores for individuals with rare variant genotypes who scored positive on the SCQ compared to individuals with idiopathic autism

|  | Idiopathic autism (n=480) | ND-CNVs_ia*  (n=252) | SGVs_ia**  (n=163) |
| --- | --- | --- | --- |
| Total score | | | |
| Mean ± SD | 27.47 ± 4.12 | 26.91 ± 3.82 | 27.68 ± 4.08 |
| Group contrast estimates (CI) | ---- | -0.58(-1.20 – 0.04) | 0.23 (-0.50 –0.97) |
| p (mixed effects linear regression)***** | ---- | 0.162 | 0.807 |
| Social subdomain score | | | |
| Mean ± SD | 13.70 ±3.25 | 13.88 ±2.92 | 14.40 ± 3.23 |
| Group contrast estimates (CI) | ---- | 0.11(-0.38 – 0.59) | 0.67 (0. 01 – 1.23) |
| p (mixed effects linear regression)***** | ---- | 0.904 | 0.055 |
| Communication subdomain score | | | |
| Mean ± SD | 7.55 ±1.63 | 7.03 ± 1.89 | 7.90 ±1.32 |
| Group contrast estimates (CI) | ---- | -0.40(-0.67 – -0.14) | 0.46 (0.17 – 0.75) |
| p (mixed effects linear regression)***** | ---- | 0.496 | **0.012** |
| Repetitive behaviour subdomain score | | | |
| Mean ± SD | 6.20 ±1.55 | 5.80 ±1.76 | 4.94 ±2.14 |
| Group contrast estimates (CI) | ---- | -0.36(-0.63 – -0.09) | -1.18 (-1.51 – -0.88) |
| p (mixed effects linear regression)***** | ---- | 0.050 | **<0.001** |

* ND-CNVs_ia: individuals with ND-CNVs who passed the cut-off of >=22 for autism on the SCQ

**SGVs_ia: individuals with SGVs who passed the cut-off of >=22 for autism on the SCQ

***p-value was corrected for multiple comparisons and remained significant following sensitivity analyses, including ethnicity, income, comorbidities, and medication use (Supplementary Tables 11, 13 and 14).

# Supplementary Table 10 Comparison of autism subdomain scores in individuals with rare variant genotypes who scored above the cutoff point (>=22) for indicative autism versus those with idiopathic autism

| **Rare variant genotypes compared to idiopathic autism** | **Group contrast estimates** | **CI** | **p (mixed effects linear regression)*** |
| --- | --- | --- | --- |
| **Social subdomain score** | | | |
| 15q11.2 deletion | 1.35 | -0.20 – 2.90 | 0.967 |
| 15q13.3 deletion | 0.27 | -1.55 – 2.09 | 1.000 |
| 16p11.2 deletion | 0.17 | -0.74 – 1.09 | 1.000 |
| 16p11.2 duplication | 0.03 | -1.00 – 1.05 | 1.000 |
| 1q21.1 deletion | -0.44 | -2.10 – 1.21 | 0.999 |
| 1q21.1 duplication | 0.43 | -0.74 – 1.59 | 0.999 |
| 22q11.2 deletion | 1.30 | -0.01 – 2.60 | 0.899 |
| 22q11.2 duplication | -0.85 | -2.55 – 0.84 | 0.899 |
| 3q29 deletion | -2.15 | -3.77 – -0.54 | 0.469 |
| ADNP | 1.91 | 0.08 – 3.73 | 0.999 |
| ASXL3 | 1.29 | -0.34 – 2.92 | 0.999 |
| DYRK1A | 0.43 | -1.39 – 2.25 | 1.000 |
| GRIN2B | 0.50 | -1.07 – 2.06 | 1.000 |
| PPP2R5D | 0.53 | -1.09 – 2.16 | 0.999 |
| STXBP1 | 0.05 | -1.36 – 1.46 | 0.999 |
| SYNGAP1 | -0.17 | -1.99 – 1.65 | 0.853 |
| SCN2A | 2.71 | 1.59 – 3.83 | **<0.001** |
| **Communication subdomain score** | | | |
| 15q11.2 deletion | -0.90 | -1.72 – -0.08 | **0.805** |
| 15q13.3 deletion | -0.56 | -1.54 – 0.42 | 0.999 |
| 16p11.2 deletion | -0.37 | -0.86 – 0.12 | 0.999 |
| 16p11.2 duplication | -0.08 | -0.62 – 0.46 | 1.000 |
| 1q21.1 deletion | -1.32 | -2.19 – -0.44 | **0.251** |
| 1q21.1 duplication | -0.19 | -0.79 – 0.40 | 0.999 |
| 22q11.2 deletion | -1.48 | -2.18 – -0.78 | **0.006** |
| 22q11.2 duplication | 0.17 | -0.71 – 1.05 | 1.000 |
| 3q29 deletion | 0.23 | -0.64 – 1.10 | 0.999 |
| ADNP | 0.99 | 0.01 – 1.97 | **0.891** |
| ASXL3 | 0.94 | 0.06 – 1.81 | **0.832** |
| DYRK1A | -0.44 | -1.42 – 0.54 | 0.379 |
| GRIN2B | 0.64 | -0.21 – 1.48 | 0.139 |
| PPP2R5D | 0.03 | -0.85 – 0.90 | 0.948 |
| STXBP1 | 0.79 | 0.03 – 1.55 | **0.043** |
| SYNGAP1 | -0.36 | -1.34 – 0.62 | 0.469 |
| SCN2A | 0.73 | 0.13 – 1.34 | **0.645** |
| **Repetitive behaviour subdomain score** | | | |
| 15q11.2 deletion | -0.37 | -1.23 – 0.49 | 0.999 |
| 15q13.3 deletion | -1.19 | -2.20 – -0.19 | **0.068** |
| 16p11.2 deletion | -0.50 | -1.01 – 0.00 | 0.904 |
| 16p11.2 duplication | -0.04 | -0.61 – 0.53 | 1.000 |
| 1q21.1 deletion | -0.48 | -1.40 – 0.43 | 0.999 |
| 1q21.1 duplication | 0.33 | -0.32 – 0.97 | 0.999 |
| 22q11.2 deletion | -1.37 | -2.09 – -0.64 | **0.028** |
| 22q11.2 duplication | 0.14 | -0.80 – 1.08 | 0.999 |
| 3q29 deletion | -0.28 | -1.17 – 0.62 | 1.000 |
| ADNP | -0.59 | -1.60 – 0.42 | 1.000 |
| ASXL3 | -0.53 | -1.43 – 0.37 | 1.000 |
| DYRK1A | -0.66 | -1.67 – 0.35 | 0.999 |
| GRIN2B | -2.43 | -3.30 – -1.56 | **<0.001** |
| PPP2R5D | -0.88 | -1.78 – 0.02 | 1.000 |
| STXBP1 | -1.49 | -2.27 – -0.71 | 0.999 |
| SYNGAP1 | -0.36 | -1.37 – 0.65 | 0.999 |
| SCN2A | -2.23 | -2.85 – -1.61 | **<0.001** |

# Supplementary Table 11 Indicative autism prevalence total and subdomain scores in rare variant genotypes after accounting for background, income, other conditions*, and medication use.

| **Indicative autism prevalence in individuals with ND-CNVs or SGVs compared to controls** | | | | | | | | | |
| --- | --- | --- | --- | --- | --- | --- | --- | --- | --- |
|  | | Controls | | ND-CNVs | | | SGVs | | ND-CNVs & SGVs |
| Individuals with completed measure | | 460 | | 1005 | | | 309 | | 1314 |
| Indicative autism prevalence n (%) | | 10 (2.2) | | 252(25.1) | | | 163 (52.3) | | 415 (31.6) |
| Odds ratio (CI) | | --- | | 15.4(3.9-61.4) | | | 71.8 (17.2-299.3) | | 30.77(4.5-212.9) |
| p (mixed effects logistic regression) | | --- | | <0.001 | | | <0.001 | | <0.001 |
| **Indicative autism prevalence in individuals with ND-CNVs compared to those with SGVs** | | | | | | | | | |
|  | | ND-CNVs | | | | SGVs | | | |
| Individuals with completed measure | | 1005 | | | | 309 | | | |
| Indicative autism prevalence n (%) | | 252(25.1) | | | | 163 (52.3) | | | |
| Odds ratio (CI) | | --- | | | | 4.13 (2.2-7.6) | | | |
| p (mixed effects logistic regression) | | --- | | | | **0.002** | | | |
| **Autism total and subdomain scores in individuals with SGVs compared to those with ND-CNVs** | | | | | | | | | |
| Autism total and subdomain scores | ND-CNVs  Mean ± SD | | SGVs  Mean ± SD | | Group contrast estimates (CI) | | | p (mixed effects linear regression) | |
| Total score | 14.9-± 8.7 | | 21.1± 8.3 | | 7.0 (4.6-9.4) | | | **<0.001** | |
| Social subdomain score | 7.3±5.2 | | 10.4±5.4 | | 3.9 (2.6-5.3) | | | **<0.001** | |
| Communication subdomain score | 4.4±2.6 | | 6.3±2.4 | | 2.0 (1.3-2.7) | | | **<0.001** | |
| Repetitive behaviour subdomain score | 3.2±2.5 | | 4.2±2.2 | | 0.9 (0.3-1.4) | | | **0.001** | |
| **Autism total and subdomain scores in individuals with SGVs compared to those with idiopathic autism** | | | | | | | | | |
| Autism total and subdomain scores | Idiopathic autism (n=480)  Mean ± SD | | SGVs_ia**  (n=163)  Mean ± SD | | Group contrast estimates (CI) | | | p (mixed effects linear regression) | |
| Total score | 27.47 ± 4.12 | | 27.68 ± 4.08 | | 0.3 (-0.49 –0.99) | | | 0.162 | |
| Social subdomain score | 13.70 ±3.25 | | 13.88 ±2.92 | | 0.7 (0. 12–1.26) | | | 0.050 | |
| Communication subdomain score | 7.55 ±1.63 | | 7.03 ± 1.89 | | 0.4 (0.2-2.6) | | | **0.024** | |
| Repetitive behaviour subdomain score | 6.20 ±1.55 | | 5.80 ±1.76 | | -1.17 (-1.49 – -0.85) | | | **<0.001** | |

*****ADHD, any anxiety, seizures, sleep problems, oppositional defiant disorder, conduct disorder, depression, tic disorder

**SGVs_ia : individuals with SGVs who passed the cut-off of >=22 for autism on the SCQ

# Supplementary Table 12 Comparison of indicative autism prevalence in individuals with rare variant genotypes and controls after accounting for FSIQ, VIQ and PIQ

| **Indicative autism prevalence in individuals with rare variant genotypes compared to controls** | | |
| --- | --- | --- |
|  | Controls | Rare variant genotypes |
| **FSIQ** | | |
| Individuals with completed measure | 319 | 691 |
| Indicative autism n (%) | 9(3) | 154 (22) |
| Odds ratio (CI) | --- | 11.0 (3.1-39.4) |
| p (mixed effects logistic regression) | --- | **<0.001** |
| **VIQ** | | |
| Individuals with completed measure | 320 | 694 |
| Indicative autism n (%) | 9(3) | 154(22) |
| Odds ratio (CI) | --- | 13.6(3.7-50.2) |
| p (mixed effects logistic regression) | --- | **<0.001** |
| **PIQ** | | |
| Individuals with completed measure | 318 | 701 |
| Indicative autism n (%) | 9(3) | 161(23) |
| Odds ratio (CI) | --- | 15.0(4.0-56.5) |
| p (mixed effects logistic regression) | --- | **<0.001** |

# Supplementary Table 13 Comparison of indicative autism prevalence, total and subdomain scores in individuals with rare variant genotypes and controls after excluding the SCQ current version.

| **Indicative autism prevalence in individuals with ND-CNVs or SGVs compared to controls** | | | | | | | |
| --- | --- | --- | --- | --- | --- | --- | --- |
|  | Controls | CNVs | | SGVs | | | ND-CNVs & SGVs |
| Individuals with completed measure | 374 | 857 | | 309 | | | 1166 |
| Indicative autism n (%) | 8(2) | 231(27.1) | | 163 (52.3) | | | 394 (33.8) |
| Odds ratio (CI) | --- | 23.8(6.3-89.2) | | 74.3 (19.2-267.3) | | | 21.6 (8.7-53.6) |
| p (mixed effects logistic regression) | --- | **<0.001** | | **<0.001** | | | **<0.001** |
| **Indicative autism prevalence in individuals with ND-CNVs compared to SGVs** | | | | | | | |
|  | ND-CNVs | | | | SGVs | | |
| Individuals with completed measure | 857 | | | | 309 | | |
| Indicative autism n (%) | 231(27.1) | | | | 163 (52.3) | | |
| Odds ratio (CI) | --- | | | | 2.85 (1.70-4.8) | | |
| p (mixed effects logistic regression) | --- | | | | **<0.001** | | |
| **Autism total and subdomain scores in individuals with SGVs compared to ND-CNVs*** | | | | | | | |
| Autism total and subdomain scores | ND-CNVs  Mean ± SD | | SGVs  Mean ± SD | | | Group contrast estimates (CI) | p (mixed effects linear regression) |
| Total score | 15.2-± 8.5 | | 18.7± 7.0 | | | 4.8 (2.4-7.3) | **<0.001** |
| Social subdomain score | 7.6±5.3 | | 10.4±5.4 | | | 2.6 (1.3-3.8) | **<0.001** |
| Communication subdomain score | 4.3±2.5 | | 4.1±1.9 | | | 1.2 (0.5-1.97) | **0.001** |
| Repetitive behaviour subdomain score | 3.4±2.5 | | 4.2±2.2 | | | 0.96 (0.29-1.6) | **0.005** |

*Sensitivity analysis was not conducted to compare those with SGVs or idiopathic autism since both groups were assessed using the lifetime version only.

# Supplementary Table 14 Comparison of indicative autism prevalence, total and subdomain scores in individuals with rare variant genotypes after excluding nonverbal individuals.

| **Indicative autism prevalence in individuals with ND-CNVs and SGVs compared to controls** | | | | | |
| --- | --- | --- | --- | --- | --- |
|  | Controls | ND-CNVs | SGVs | ND-CNVs & SGVs | |
| Individuals with completed measure | 456 | 925 | 133 | 1058 | |
| Indicative autism n (%) | 9 (2) | 211 (23) | 47 (35) | 258 (24) | |
| Odds ratio (CI) | --- | 22.7 (6.4-809.0) | 62.3 (15.9-243.2) | 30.9 (7.3-131.1) | |
| p (mixed effects logistic regression) | --- | **<0.001** | **<0.001** | **<0.001** | |
| **Indicative autism prevalence in individuals with ND-CNVs compared to SGVs** | | | | | |
|  | ND-CNVs | | | SGVs | |
| Individuals with completed measure | 925 | | | 133 | |
| Indicative autism n (%) | 211 (23) | | | 47 (35) | |
| Odds ratio (CI) | --- | | | 2.5 (1.3-4.9) | |
| p (mixed effects logistic regression) | --- | | | **0.006** | |
| **Autism total and subdomain scores individuals with SGVs compared to ND-CNVs** | | | | | |
| Autism total and subdomain scores | ND-CNVs  Mean ± SD | SGVs  Mean ± SD | | Group contrast estimates (CI) | p (mixed effects linear regression) |
| Individuals with completed measure | 925 | 133 | | -- | -- |
| Total score | 14.5± 8.5 | 17.8± 7.7 | | 4.6 (3.14-6.1) | **<0.001** |
| Social subdomain score | 7.1±5.1 | 8.4±5.0 | | 2.0 (1.1-2.9) | **<0.001** |
| Communication subdomain score | 4.3±2.6 | 5.3±2.2 | | 1.3 (0.8-1.7) | **<0.001** |
| Repetitive behaviour subdomain score | 3.1±2.5 | 4.1±2.3 | | 1.3 (0.9-1.7) | **<0.001** |
| **Autism total and subdomain scores in individuals with SGVs compared to idiopathic autism** | | | | | |
| Autism total and subdomain scores | Idiopathic autism (n=421)  Mean ± SD | | SGVs_ia*  (n=47)  Mean ± SD | Group contrast estimates (CI) | p (mixed effects linear regression) |
| Total score | 27.06± 3.90 | | 26.43 ± 3.30 | -0.47 (-1.11 – 0.18) | 0.155 |
| Social subdomain score | 13.52 ±3.22 | | 13.49 ±2.52 | 0.23 (-0.28 – 0.75) | 0.377 |
| Communication subdomain score | 7.35 ±1.63 | | 7.23 ± 140 | -0.11 (-0.64 – 2.6) | **0.024** |
| Repetitive behaviour subdomain score | 6.19±1.53 | | 5.70 ±1.74 | -0.40 (-0.67 – 0.13) | 0.177 |

*SGVs_ia: individuals with SGVs who passed the cut-off of >=22 for autism on the SCQ

**References**

1. Cunningham AC, Hall J, Einfeld S, Owen MJ, van den Bree MBM. Assessment of emotions and behaviour by the Developmental Behaviour Checklist in young people with neurodevelopmental CNVs. *Psychol Med* 2022; **52**(3): 574-86.

2. Cunningham AC, Hall J, Owen MJ, van den Bree MBM. Coordination difficulties, IQ and psychopathology in children with high-risk copy number variants. *Psychol Med* 2021; **51**(2): 290-9.

3. Chawner SJRA, Owen MJ, Holmans P, et al. Genotype–phenotype associations in children with copy number variants associated with high neuropsychiatric risk in the UK (IMAGINE-ID): a case-control cohort study. *The Lancet Psychiatry* 2019; **6**(6): 493-505.

4. Wolstencroft J, Wicks F, Srinivasan R, et al. Neuropsychiatric risk in children with intellectual disability of genetic origin: IMAGINE, a UK national cohort study. *The Lancet Psychiatry* 2022; **9**(9): 715-24.

5. Pollak RM, Zinsmeister MC, Murphy MM, Zwick ME, the Emory 3q P, Mulle JG. New phenotypes associated with 3q29 duplication syndrome: Results from the 3q29 registry. *American Journal of Medical Genetics Part A* 2020; **182**(5): 1152-66.

6. Glassford MR, Rosenfeld JA, Freedman AA, Zwick ME, Mulle JG, Unique Rare Chromosome Disorder Support G. Novel features of 3q29 deletion syndrome: Results from the 3q29 registry. *American journal of medical genetics Part A* 2016; **170A**(4): 999-1006.

7. Seitz-Holland J, Lyons M, Kushan L, et al. Opposing white matter microstructure abnormalities in 22q11.2 deletion and duplication carriers. *Transl Psychiatry* 2021; **11**(1): 580.

8. The Simons Vip Consortium. Simons Variation in Individuals Project (Simons VIP): A Genetics-First Approach to Studying Autism Spectrum and Related Neurodevelopmental Disorders. *Neuron* 2012; **73**(6): 1063-7.
