# Supplementary material for: Comparison of autism domains across thirty rare variant genotypes
Source: eBioMedicine. 2025 Jan 31;112:105521. doi: 10.1016/j.ebiom.2024.105521 (PMC11835590; doi:10.1016/j.ebiom.2024.105521)
Supplement: IMAGINE ID Consortioum Members [file mmc2.docx]

**Appendix: IMAGINE ID Consortium members**

| **Surname** | **Initials** | **First Name** | **Title** | **Institution** |
| --- | --- | --- | --- | --- |
| Housby | H | Harriet | Ms | Great Ormond Street Institute of Child Health, University College London, UK |
| Lee | I | Irene | Mrs | Great Ormond Street Institute of Child Health, University College London, UK |
| Skuse | D | David | Professor | Great Ormond Street Institute of Child Health, University College London, UK |
| Wolstencroft | J | Jeanne | Dr | Great Ormond Street Institute of Child Health, University College London, UK |
| Mandy | W | William | Dr | Division of Psychology & Language Sciences, University College London, UK |
| Denaxas | S | Spiros | Dr | Institute of Health Informatics, University College London, London, UK |
| Baker | K | Kate | Dr | Department of Medical Genetics, University of Cambridge, UK |
| Raymond | FLF | Lucy | Professor | Department of Medical Genetics, University of Cambridge, UK |
| van den Bree | MBM | Marianne | Professor | MRC Centre for Neuropsychiatric Genetics and Genomics, Division of Psychological Medicine and Clinical Neurosciences, Cardiff University, UK |
| Chawner | SJRA | Samuel | Dr | MRC Centre for Neuropsychiatric Genetics and Genomics, Division of Psychological Medicine and Clinical Neurosciences, Cardiff University, UK |
| Hall | J | Jeremy | Professor | MRC Centre for Neuropsychiatric Genetics and Genomics, Division of Psychological Medicine and Clinical Neurosciences, Cardiff University, UK |
| Holmans | P | Peter | Professor | MRC Centre for Neuropsychiatric Genetics and Genomics, Division of Psychological Medicine and Clinical Neurosciences, Cardiff University, UK |
| Hope-Bell | J | Josh | Dr | MRC Centre for Neuropsychiatric Genetics and Genomics, Division of Psychological Medicine and Clinical Neurosciences, Cardiff University, UK |
| Le Roux | D | Danielle | Ms | MRC Centre for Neuropsychiatric Genetics and Genomics, Division of Psychological Medicine and Clinical Neurosciences, Cardiff University, UK |
| Morrin | S | Sally | Ms | MRC Centre for Neuropsychiatric Genetics and Genomics, Division of Psychological Medicine and Clinical Neurosciences, Cardiff University, UK |
| Owen | MJ | Michael | Professor Sir | MRC Centre for Neuropsychiatric Genetics and Genomics, Division of Psychological Medicine and Clinical Neurosciences, Cardiff University, UK |
| Sivakumar | S | Shreeya | Ms | MRC Centre for Neuropsychiatric Genetics and Genomics, Division of Psychological Medicine and Clinical Neurosciences, Cardiff University, UK |
